# Temporal dynamics of a CSF1R signaling gene regulatory network involved in epilepsy

**DOI:** 10.1371/journal.pcbi.1008854

**Published:** 2021-04-05

**Authors:** Claude Gérard, Laurane De Mot, Sabine Cordi, Jonathan van Eyll, Frédéric P. Lemaigre

**Affiliations:** 1 Université catholique de Louvain, de Duve Institute, Brussels, Belgium; 2 Clarivate Analytics, Carlsbad, California, United States of America; 3 UCB Pharma, Braine-l’Alleud, Belgium; University of Pittsburgh, UNITED STATES

## Abstract

Colony Stimulating Factor 1 Receptor (CSF1R) is a potential target for anti-epileptic drugs. However, inhibition of CSF1R is not well tolerated by patients, thereby prompting the need for alternative targets. To develop a framework for identification of such alternatives, we here develop a mathematical model of a pro-inflammatory gene regulatory network (GRN) involved in epilepsy and centered around CSF1R. This GRN comprises validated transcriptional and post-transcriptional regulations involving STAT1, STAT3, NFκB, IL6R, CSF3R, IRF8, PU1, C/EBPα, TNFR1, CSF1 and CSF1R. The model was calibrated on mRNA levels of all GRN components in lipopolysaccharide (LPS)-treated mouse microglial BV-2 cells, and allowed to predict that STAT1 and STAT3 have the strongest impact on the expression of the other GRN components. Microglial BV-2 cells were selected because, the modules from which the GRN was deduced are enriched for microglial marker genes. The function of STAT1 and STAT3 in the GRN was experimentally validated in BV-2 cells. Further, *in silico* analysis of the GRN dynamics predicted that a pro-inflammatory stimulus can induce irreversible bistability whereby the expression level of GRN components occurs as two distinct states. The irreversibility of the switch may enforce the need for chronic inhibition of the CSF1R GRN in order to achieve therapeutic benefit. The cell-to-cell heterogeneity driven by the bistability may cause variable therapeutic response. In conclusion, our modeling approach uncovered a GRN controlling CSF1R that is predominantly regulated by STAT1 and STAT3. Irreversible inflammation-induced bistability and cell-to-cell heterogeneity of the GRN provide a theoretical foundation to the need for chronic GRN control and the limited potential for disease modification via inhibition of CSF1R.

## Author summary

Epilepsy is associated with the induction of complex molecular inflammatory processes. A better understanding of these molecular mechanisms is crucial to optimize therapeutic options. Here, we identified a gene regulatory network (GRN) involved in epilepsy that is controlled by inflammation and which regulates the expression and function of Colony Stimulating Factor 1 receptor (CSF1R), a therapeutic target for anti-epileptic drugs. Using mathematical modeling and experiments with cultured cells, we found that two of eleven components of the network, namely STAT1 and STAT3, exert a tight control on all other components. In addition, we found that inflammation can induce an irreversible switch in the expression of all components of the network, and can cause high cell-to-cell variability. Our findings provide a framework explaining why chronic, not acute, anti-inflammatory treatment is necessary to modulate the network and why drugs targeting CSF1R have limited therapeutic potential.

## Introduction

Epilepsy is a heterogeneous disease characterized by recurrent unprovoked seizures, cognitive and behavioral impairments, and increased risk of death [1]. Despite the availability of diverse therapeutic options [2], a third of patients do not show clinical benefit from current anti-epileptic drugs (AEDs). Also, epileptic syndromes result from multiple causes, yet comprehensive mechanisms of epilepsies as well of AED refractoriness remain poorly understood [2,3].

Chronic neuroinflammation is a common biological feature of epilepsies and inhibition of the inflammatory response has demonstrated therapeutic efficacy in preclinical contexts [4–6]. In this context, our previous study identified Colony Stimulating Factor 1 receptor (CSF1R) as a new therapeutic target, as its inhibition induced anti-inflammatory effects associated with a decrease in epileptic seizures in a pilocarpine mouse model of epilepsy [6]. Despite encouraging preclinical results, the clinical side effects of long-term inhibition of CSF1R are not acceptable for epileptic patients [7]. Therefore, this treatment could only be administered in an acute protocol. However, other studies on microglia indicate that the effects of CSF1R inhibition require long-term exposure to inhibitors and are transient [8–12]. Nevertheless, long-term exposure is associated with hepatoctoxicity and depletion of microglia and of circulating monocytes. Therefore, identifying a therapeutic target that shares the beneficial consequences of CSF1R inhibition on neuro-inflammation, but devoid of side effects, would be of significant interest.

To address this challenge we reasoned that CSF1R controls a gene regulatory network (GRN) which may comprise alternative entry points for therapy. We defined the structure of a GRN composed of transcriptional and post-translational regulations involving *Csf1r*, *Csf1*, *Stat1*, *Stat3*, *Irf8*, *Nfkb*, *Pu1*, *Il6R*, *Csf3r*, *Tnfr1* and *Cebpa*. A mathematical model was developed to assess the relative influence of individual network component on the GRN dynamics. It predicted that *Stat1*, *Stat3*, and *Cebpa* are key modulators of the other network members, and this was validated experimentally for *Stat1* and *Stat3* by siRNA-mediated inhibition in BV-2 cells. Moreover, model-based simulations of the dynamics of the GRN revealed that it is characterized by irreversible bistability and strong cell-to-cell heterogeneity, suggesting that therapeutic modulation of the CSF1R-regulating GRN would require long-term and chronic exposure to drugs targeting this GRN.

## Results

### Identification of an inflammation-regulated gene regulatory network involving CSF1R

In earlier work, we and others identified and modeled GRNs based on a set of stringent functional criteria where GRN members are connected by direct functional links characterized by protein-protein and protein-DNA interactions, or epistatic relationships identified in loss- and gain- of function analyses. In that context, in our earlier work we selected literature data that provide solid and well argumented conclusions about functional interactions between GRN members to design the network structure or the dynamics of several signalling pathways [13–15]. Here we adapted this strategy by analysing interactions between the GRN components which were obtained from the Clarivate Analytics MetaBase (version 6.15.62452, https://clarivate.com/products/metacore/), which is a meta-database of manually curated literature-based contextual biological interactions. To build the network and identify the nearest neighbours of CSF1R, we used logical criteria to filter the GRN in order to reduce its size.

More precisely, to identify the structure of a CSF1R-regulating GRN in epilepsy, we took advantage of a set of 6 co-expression gene modules which were previously predicted (and, for one of them, validated) to be activated by this receptor. The gene modules were initially identified from bulk sequencing of RNA extracted from hippocampus in a mouse model of pilocarpine-induced epilepsia, and then found to be conserved between mouse and human [6]. These modules are also differentially co-expressed between control and disease state, and 5 of them significantly correlate with seizure frequency. Moreover, these modules are enriched for functional terms related to immune response and/or inflammatory processes, or for microglial or astrocyte marker genes, indicating their role in the establishment of neuro-inflammation in the context of epilepsy pathogenesis.

**Fig 1:**
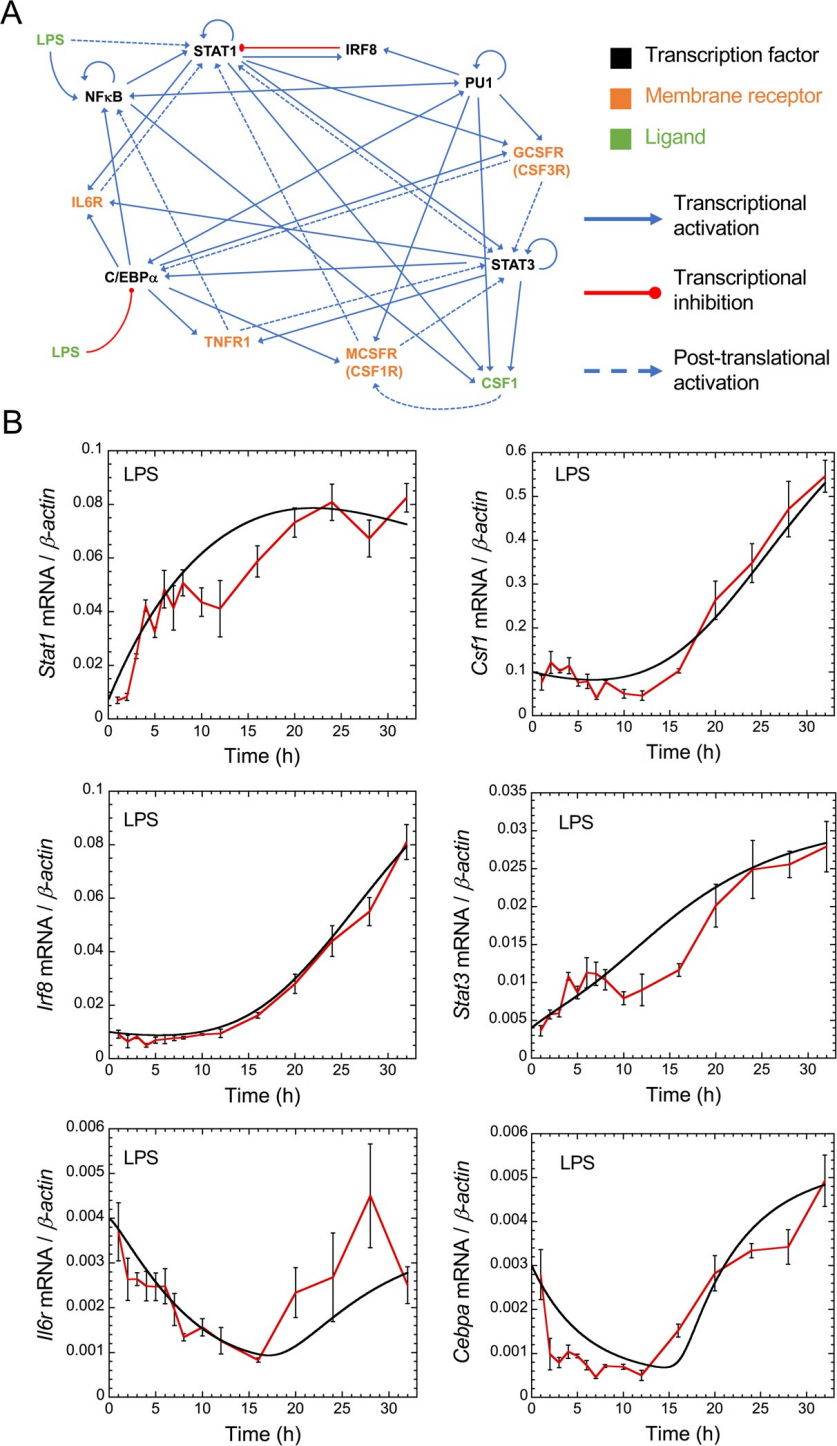
Structure and temporal dynamics of the gene regulatory network. (A) Structure of the GRN. (B) Temporal evolution of the mRNA expression levels of *Stat1*, *Csf1*, *Irf8*, *Stat3*, *Il6r* and *Cebpa* in the presence of LPS (1 μg/ml added at t = 0h). Red curves: experimental data, black curves: mathematical model. Experimental data (S1 and S2 Data) are means (relative to β-actin) +/- SD and were collected at the indicated time points after LPS administration in BV-2 cells. Conditions of the numerical simulations are described in S1 Text and Tables A-E in S1 Tables.

A GRN centered around CSF1R was derived from these 6 co-expression gene modules and from their predicted cross-regulations [6]. The initial set of nodes included in the GRN was selected from the 96 regulators (75 cell membrane proteins and 21 transcription factors) whose genes are part of a module and were predicted to be significant regulators of at least one module. We applied multiple filtering analysis to reduce the complexity of the network and to obtain a GRN amenable to mathematical modeling, as described in the Methods section and S1–S5 Figs. The final GRN comprises 11 components: 1 ligand (CSF1), 6 transcription factors (STAT1, STAT3, IRF8, PU1, NFκB and C/EBPα) and 4 cell-membrane receptors (IL6R, TNFR1, CSF1R and CSF3R) (Fig 1A).

### Calibration of the mathematical model

To characterize the dynamics of the GRN identified above and to identify key components of the network, a mathematical model of the GRN based on a set of kinetic equations was built. The model describes the temporal evolution of the mRNA levels of all network components, and of the inactive and active protein forms of the component that are post-translationally regulated, namely STAT1, STAT3, CSF1R, NFκB and C/EBPα. The addition of an activation or inactivation term determines the switch from inactive to active, or from active to inactive form of the protein. We assumed that proteins were expressed at a level 3000-fold higher than the corresponding mRNA, according to Schwanhausser et al., who showed that the mean protein/mRNA ratio for 5000 different genes is close to 3000 [16]. The detailed description of the model, the definition of the variables and the kinetic equations are provided in S1 Text and Tables A-C in S1 Tables.

To calibrate the mathematical model, we used RT-qPCR to measure the mRNA level of each network component in mouse BV-2 cells, in the absence (S6 Fig) or in the presence of lipopolysaccharide (LPS), during 32h (Figs 1B and S7). LPS was used to activate an inflammatory response in BV-2 cells resulting in an induction mRNA expression of all GRN components except for *Il6r* and *Cebpa* which are transiently downregulated. The results showed that the majority of experimental values fitted semi-quantitatively the *in silico* predicted mRNA levels of each network component. In particular, the model reproduces the temporal dynamics of each component which is very heterogeneous. Indeed, *Stat1* and *Stat3* mRNA expression increase quickly after LPS administration (Fig 1B); *Csf1*, *Irf8* and *Tnfr1* mRNA expression rise only after a delay (Figs 1B and S7), *Nfkb* mRNA expression increases rapidly but transiently (S7 Fig), while *Il6r* and *Cebpa* mRNA expression decrease transiently after LPS administration (Fig 1B).

### *In silico* prediction and experimental validation of the key components of the network

**Fig 2:**
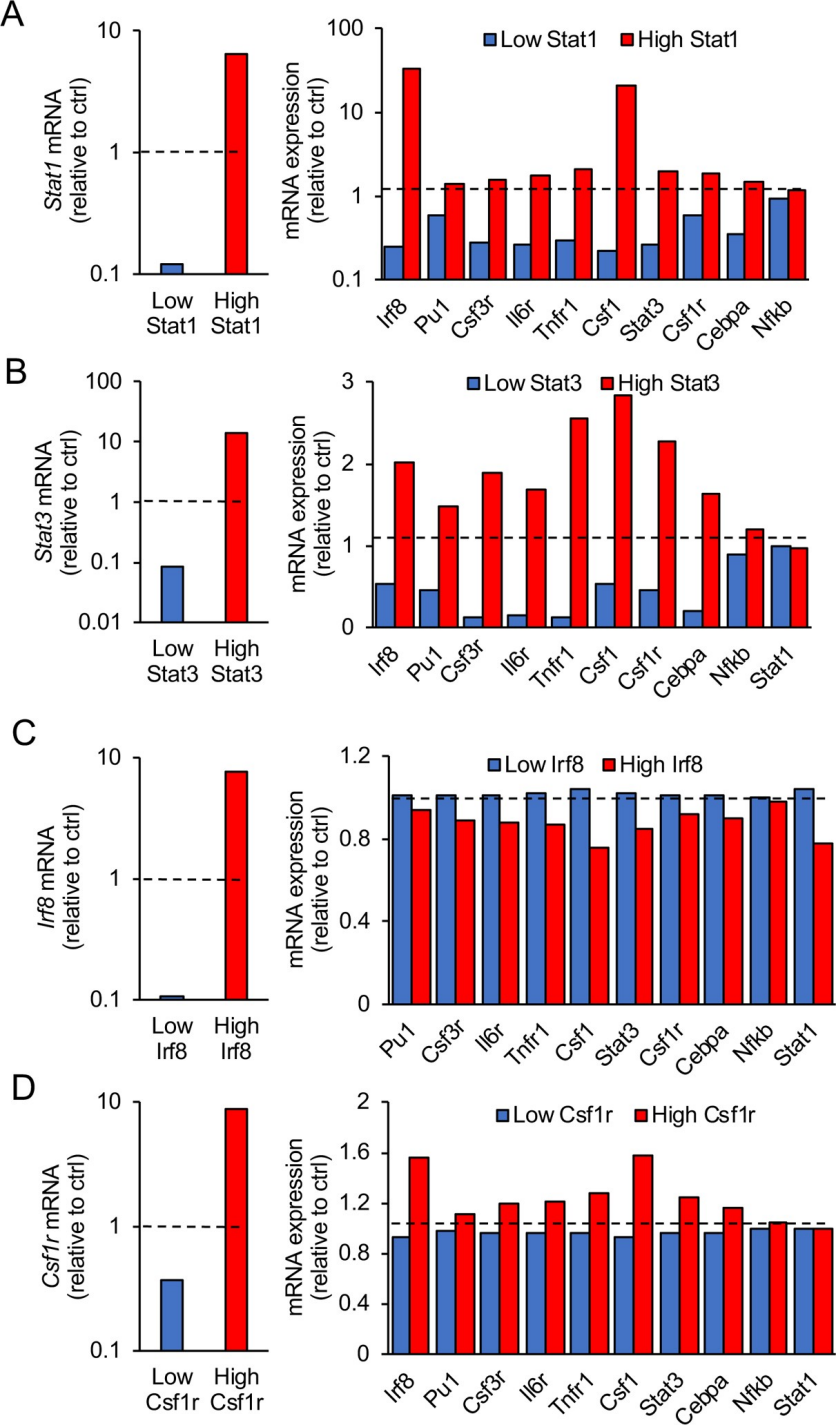
*In silico* prediction of the impact of *Stat1*, *Stat3*, *Irf8* and *Csf1r* expression levels on the GRN dynamics. Impact of low (blue bars) or high expression levels (red bars) of *Stat1* (A), *Stat3* (B), *Irf8* (C) and *Csf1r* (D) on the mRNA expression of the other GRN components after 48h. (A, B) *Stat1* and *Stat3* are predicted to have a strong impact on the mRNA expression of the network components, unlike *Irf8* and *Csf1r* (C, D). The control conditions in the simulations (horizontal dashed lines) are the expression levels of the GRN components in the absence of LPS after 48h. Numerical simulations for the various conditions are described in S1 Text and Tables A-E in S1 Tables.

To predict the impact of each component on the expression of the others, a 5- to 10-fold increase or decrease in expression of all individual mRNAs was simulated *in silico*. The outcome suggested that *Stat1*, *Stat3* and *Cebpa* strongly impact the mRNA levels of the other network components, while *Irf8*, *Csf1r*, *Pu1*, *G-Csfr*, *Il6r*, *Tnfr1*, *Nfkb*, and *Csf1* were predicted to have limited influence (Figs 2 and S8 and S9).

**Fig 3:**
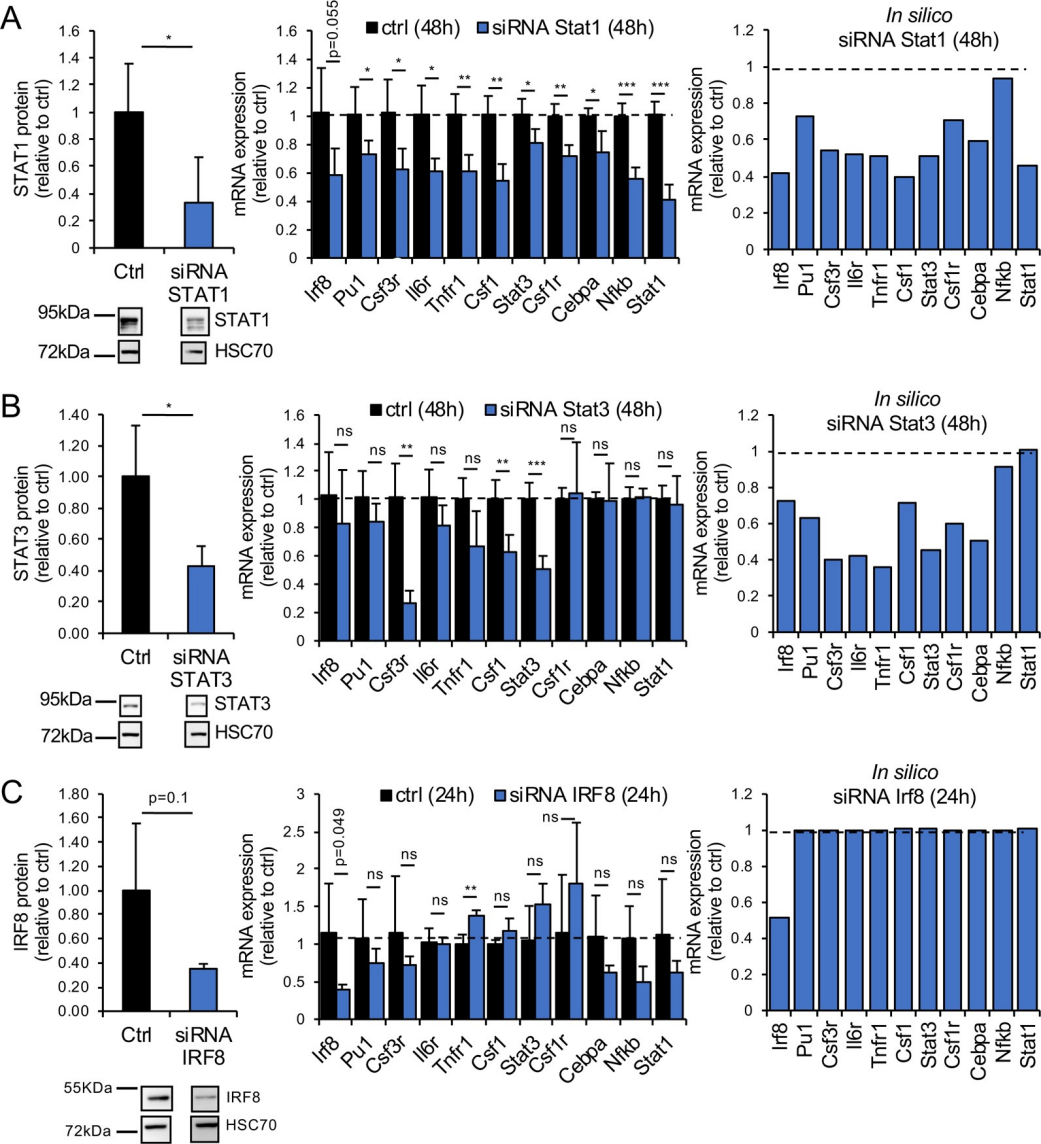
Experimental validation of the mathematical model. Transient siRNA-mediated inhibition of *Stat1*, *Stat3* and *Irf8* in BV-2 cells downregulates the expression of the corresponding proteins (left panels in A-C). Downregulation of STAT1 and STAT3 strongly impacts the mRNA expression of the GRN components, unlike downregulation of IRF8 (middle panels in A-C; S3 Data). *In silico* simulations of the consequences of STAT1, STAT3 and IRF8 downregulation on the mRNA expression levels of the network components are in agreement with experimental data (compare middle and right panels in A-C). Protein expression levels are first normalized to HSC70 expression and then to the expression of the protein of interest (STAT1, STAT3 or IRF8) in the control condition (ctrl-siRNA treatment). Similarly, mRNA expression levels are first normalized to *β-actin* expression and then to the gene expression level in the control condition (2-ΔΔCT). In the simulations, the control condition is the expression of the GRN components in the absence of LPS after 48h (A, B) or 24h (C). 24h or 48h of LPS treatments are the minimum time duration required to observe an impact on the expression levels of the GRN components. Numerical simulations for the various conditions are described in S1 Text and Tables A-E in S1 Tables. Data are means ± SD; n ≥ 4, *, p<0.05; **, p<0.01.

To validate the model, we compared the *in silico* predicted impact of *Stat3*, *Stat1* and *Irf8* downregulation with the results obtained in BV-2 cells transiently transfected with siRNAs (Fig 3). Anti-*Stat1* siRNA treatment downregulated the expression of STAT1 protein and *Stat1* mRNA by more than 50%, and reduced the mRNA levels of all other GRN components (Fig 3A). Except for *Nfkb*, the model correctly predicted that a 50% inhibition of *Stat1* decreased the mRNA expression levels of all other network components (compare middle and right panels in Fig 3A). Importantly *in silico* predictions predicted the experimental data at the appropriate time scale. Similarly, anti-*Stat3* siRNA downregulated STAT3 protein and mRNA by 50%, leading to reduced expression of several other network members (Fig 3B). The *in silico*-predicted impact of STAT3 inhibition on the expression of mRNA of the other network components fitted most experimental data (compare middle and right panels in Fig 3B). Despite several attempts, anti-*Cebpa* siRNA treatment was not able to affect the expression level of the GRN components, likely as a result of the low expression of *Cebpa* in BV-2 cells (Fig 1B). Finally, as predicted by the mathematical model, a siRNA-induced decrease in IRF8 did not influence the mRNA levels of the other network components (Fig 3C). Thus, we concluded that STAT1 and STAT3 are key regulators of the here-defined CSF1R-regulating GRN.

### The CSF1R network dynamics exhibit bistability

**Fig 4:**
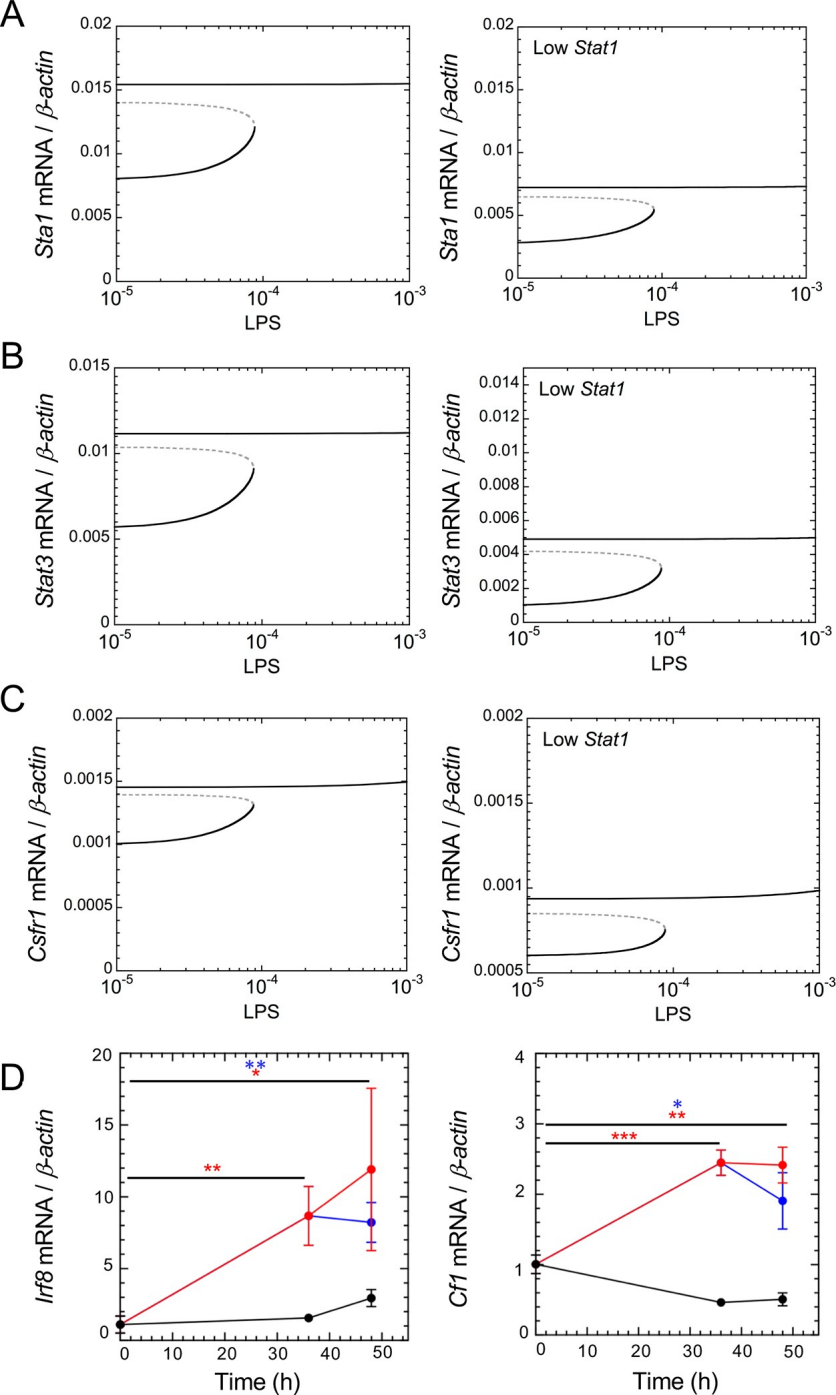
LPS-induced irreversible bistability in the CSF1R-regulating GRN. mRNA steady-state levels of *Stat1* (A), *Stat3* (B) and *Csf1r* (C) are shown as a function of LPS in the basal condition (left panels) or with 50% of *Stat1* downregulation (right panels). Black curves: stable steady states, gray dashed curves: unstable steady states. Determination of bifurcation diagrams for the different conditions is described in S1 Text and Tables A-E in S1 Tables. (D) BV2 cells were treated with LPS for 36h followed by an additional 12h period in the presence or absence of LPS (S4 Data). The data (means ± SD; *, p<0.05; **; p<0.01; ***, p< 0.001; n = 3 to 4) show the expression of *Irf8* and *Csf1*, which were selected because of their short half-life.

Next, the CSF1R network dynamics were studied using the experimentally-validated mathematical model. Steady-state expression levels of *Stat1*, *Stat3* and *Csf1r* mRNA plotted as a function of LPS indicate that irreversible bistability is observed at low levels of LPS (Fig 4A–4C, left panels). At high levels of LPS, only the upper stable state of the network component expression is maintained, which we define as the “GRN-activated” state. Biologically speaking LPS values must be positive, but simulations with negative values of LPS show that middle and upper branch connect in a saddle-node. The existence of irreversible bistability indicates that the CSF1R-regulating GRN, once activated by pro-inflammatory stimuli, may be locked in an activated stable steady state even after removal of stimulus (LPS in our model). The model also predicts that a 50% decrease of *Stat1* mRNA downregulates the expression of other network components, without affecting the occurrence of the bistability (compare right and left panels in Fig 4A–4C).

To support that the cells are operating in a bistable regime, we treated the cells for 36h with LPS, and replaced the medium for an additional 12h in the presence or absence of LPS. The experiment must be arrested at 48h because beyond that stage we noticed signs of LPS-induced toxicity such as morphological changes and cell mortality. We measured the expression of the *Irf8* and *Csf1* mRNAs. They were selected because they are the GRN members with the shortest half-life (4.1 and 5.2 h, respectively; Table E in S1 Tables) and because their half-life is shorter than the 12h period in the absence of LPS. The results are shown in a Fig 4D: after 12h removal of LPS, the expression of *Irf8* and *Csf1* remained high and did not differ statistically from the expression in conditions where LPS was maintained during the 12h. This experimental data supports that the transition to the active state is irreversible.

Heterogeneity plays an important role in the dynamics of the network. To illustrate this aspect we simulated the temporal evolution of Stat1 mRNA expression, in the absence or presence or LPS, and with varying initial conditions (S10 Fig). Depending on the initial conditions, Stat1 mRNA expression displays different temporal profiles, as a consequence of the bistable nature of the GRN.

**Fig 5:**
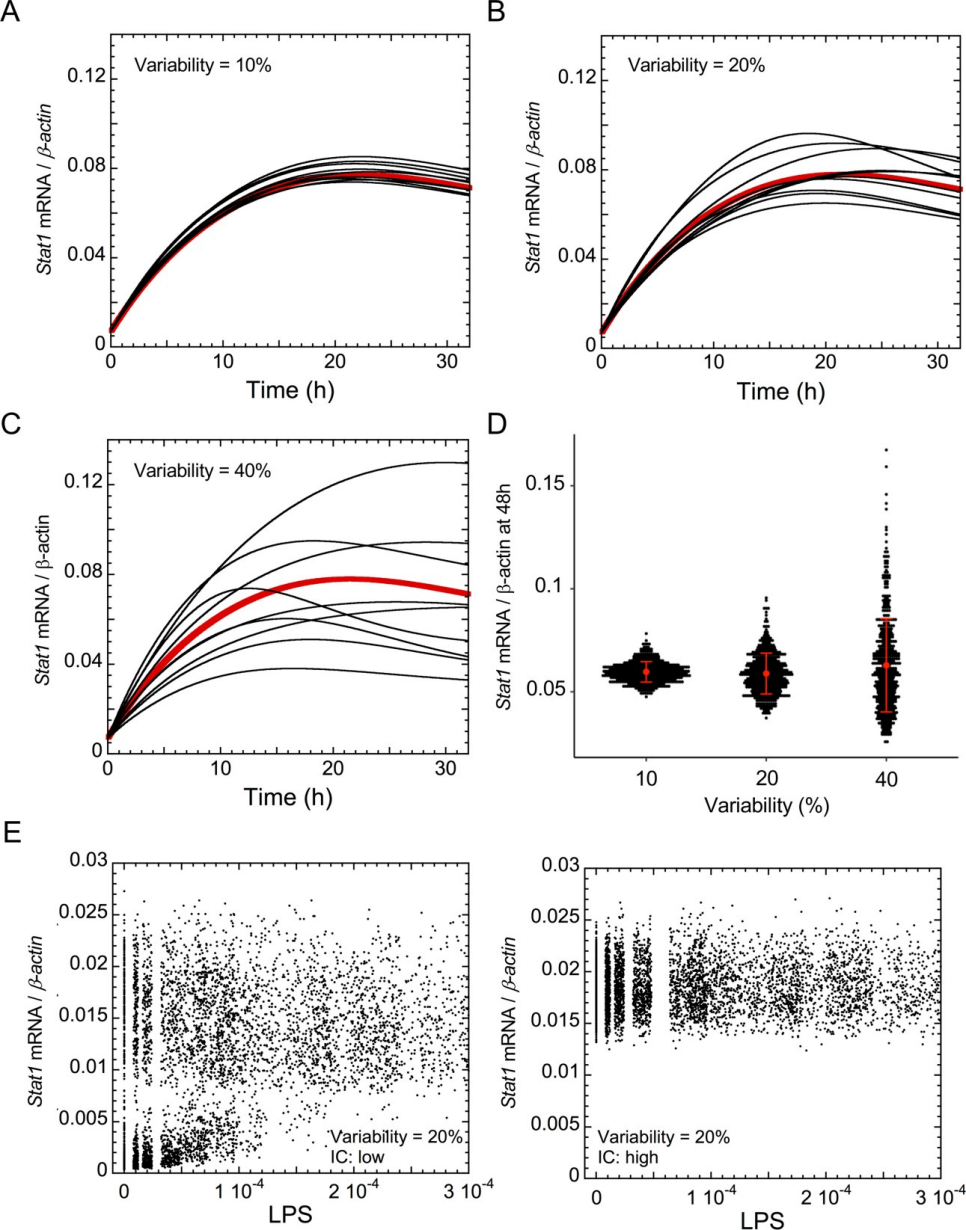
Heterogeneity of the dynamics of the CSF1R-regulating GRN. (A-C) Time series of *Stat1* mRNA in a heterogeneous cell population in the presence of LPS (LPS = 1), where 10%, 20% or 40% of uniform random variation is considered around the basal value of each parameter, for all parameters of the model. Black curves: temporal evolution of *Stat1* mRNA in one cell in the heterogeneous cell population, red curves: temporal evolution of *Stat1* mRNA in the absence of random variation. (D) *Stat1* mRNA expression after 48h of LPS treatment with 10%, 20% and 40% of uniform random variations on the parameters. Each black dot represents one cell in a heterogeneous population of 500 cells. Red dots: mean expression levels of *Stat1* mRNA +/- SEM. (E) Steady state expression levels of *Stat1* mRNA in a heterogeneous cell population for different levels of LPS in the presence of 20% of uniform random variations on all parameter values. These expression levels are shown starting from a low initial (initial condition low; left panel) or a high expression level of the GRN components (initial condition: high; right panel). The steady state was measured after 200 h of transients. Numerical simulations for the different conditions are described in S1 Text and Tables A-E in S1 Tables.

The irreversible bistability might generate cell-to-cell heterogeneity in the expression of the network components, with some cells remaining in the basal state, while others having switched to the “GRN-activated” state. To assess such heterogeneity, we performed numerical simulations in a heterogeneous cell population where uniform random variations are applied to the value of each parameter of the model (Fig 5). *Stat1* mRNA kinetics were calculated based on simulations for a heterogeneous cell population of 500 cells in the presence of 10%, 20% or 40% of random variations from the basal value of all parameters of the model (Fig 5A–5C). The model indicates that *Stat1* mRNA expression after 48h of LPS treatment exhibited a large level of variability from cell-to-cell in a cell population (Fig 5D; each black dot represents an individual cell).

The steady-state levels of *Stat1* mRNA in a heterogeneous cell population, in the presence of 20% of uniform random variations of the parameters, were also evaluated for different levels of LPS (Fig 5E). Our analysis showed that a cell population initially characterized by high expression of the network components, will remain in the activated stable steady state of the irreversible bistability, whatever the LPS concentration (Fig 5E, right panel). We consider that both stable steady states of the bistable switch are robust, with a clear transition between the bottom and the upper states of the switch, up until 20% at least of uniform random variation on all parameter values of the model. However, when cells initially defined by low levels of the network components are treated with LPS, the two cell states may occur at low LPS stimulation, and a single cell state with large heterogeneity is predicted at higher LPS stimulation (Fig 5E, left panel). Thus, the model suggests that, as a consequence of an irreversible transition, the network displays wide cell-to-cell heterogeneity and that the initial expression level of the network members will define the GRN state in the absence or in the presence of low level of inflammation.

## Discussion

We identified and characterized the dynamics of a CSF1R-regulating GRN in epilepsy and validated STAT1 and STAT3 as main regulators of this GRN. Our analyses suggest that inflammation induces bistability in the expression of all network components. Positive feedback loops likely contribute to the bistability, and several are present in the GRN. Examples include feedback loops involving IL6R (IL6R→STAT1→IL6R), NFκB (NFκB→STAT1→STAT3→C/EBPα→NFκB), or STAT3 (STAT3→C/EPBα→PU1→STAT3). Interestingly, a GRN comprising members of the presently studied network was modeled in the context of cytokine-induced differentiation of granulocyte-monocyte progenitor cells; the dynamics were also characterized by multiple stable states [17].

Our model inevitably suffers from a number of limitations. Whereas the majority of the experimental data fitted the *in silico* predictions, we could not obtain a good experimental fit with all predictions. This results at least in part from the fact that we estimated but did not measure the expression levels and activation state of proteins. Also, the structure of the network is unlikely to be complete, and other regulators which may influence the quantitative expression of the GRN components may have been missed.

Previous studies showed that selective inhibition of the JAK/STAT pathway reduces the severity of chronic epilepsy in rodents by decreasing downstream targets of STAT3 [6]. This supports the idea that shared therapeutic molecular mechanisms may be triggered by different upstream regulators, but their respective specificities could provide opportunities to minimize side effects and unwilled consequences. Unfortunately, amongst the regulators identified in the CSF1R-regulating GRN, few display a propensity to be suitable therapeutic targets in epilepsy, either because no drug crossing the blood-brain barrier are available, or because their safety profile and lack of specificity would hamper their use in chronic administration.

Our modeling approach suggests that the CSF1R-regulating network displays irreversible bistability and, as a consequence, that a cell sub-population would adopt a permanently activated GRN state after a first pro-inflammatory stimulus, even if this stimulus resolves. Also, our modeling suggests that inhibition of STAT1 does not affect the bistablity of the GRN induced by inflammation. The existence of the stable activated state could explain the need to chronically treat this pro-inflammatory GRN, as well as the observed lack of relevance of inflammation resolution in brain microglia (reviewed in [4]). We note that immunomodulation may not be causal to epilepsy but rather an amplifying bystander effect, consequent to prolonged seizures. Also, we do not claim that the CSF1R pathway is the only contributor in neuro-inflammation as others have already been extensively validated in epilepsy, including IL-1β signaling [4]. The fact is that CSF1R inhibition is one of the rare immune pathway whose modulation has demonstrated therapeutic benefit on epilepsy pre-clinical models. To rely on these pre-existing foundations of therapeutic efficacy, the GRN was arbitrarily centered around a validated target, namely CSF1R.

The potential existence of cell subpopulations might be also at the origin of a differential sensitivity to therapeutic treatment, since efficacy of a therapeutic inhibitor is expected to depend on the expression of its target. In the case of CSF1R, the cells displaying an activated GRN state can be dampened by chronic inhibition. However, as a consequence of stochastic fluctuations in the expression levels of the GRN components, a pool of basal state cells can repopulate the activated state upon treatment termination [8]. In epilepsy, seizures seem to be sufficient to induce a pro-inflammatory reaction and would explain the vicious circle between seizures and neuro-inflammation [5]. Therefore, identifying alternative GRN(s) of therapeutic interest, but not characterized by a bistable state, could provide more realistic opportunities for disease-modifying, acute treatment protocols and, potentially curative for neuro-inflammatory cascades in the epileptic brain.

In conclusion, our modeling strategy identified the dynamics of a CSF1R-regulating GRN that is mainly controlled by STAT1 and STAT3. As a result of the irreversible bistability induced by inflammation and of the cell-to-cell heterogeneity, chronic anti-inflammatory control of the GRN would be needed. Yet, our analysis suggests that drugs targeting CSF1R would have limited disease-modifying potential.

## Methods

### Identification of the GRN structure

Our identification of the GRN relies on the work of Srivastava and coworkers [6]. Briefly, this work used transcriptomic data from epileptic and control mice to assemble modules of genes that are co-expressed in the context of epilepsy. These modules were then submitted to the CRAFT methodology (“Causal Reasoning Analytical Framework for Target discovery”), which identifies upstream regulators significantly activating or inhibiting each module. These regulators are transcription factors (TFs) or cell membrane receptors (CMPs) that regulate the genes in the modules through linear canonical pathways. We updated the CRAFT analysis performed by Srivastava and coworkers with a recent version of the database of known linear pathways from MetaBase. Six modules were selected that were predicted to be regulated by CSF1R and differentially co-expressed between control and epileptic state. They correspond to modules 5o, 12o, 16o, 18o, 22o and 24o identified previously [6].

The initial set of nodes included in the GRN was selected from the 96 regulators, namely 75 CMPs and 21 TFs whose genes belong to one of these 6 modules and were predicted to be significant regulators of at least one of these modules. This ensures that the regulations used are contained in the 6 modules. CMPs encoded by several genes were included in the GRN when at least one of the coding genes belongs to a module. To account for potential intercellular signaling, 19 ligands of the 75 identified receptors were added based on MetaBase interaction database (selecting interactions with mechanism as “receptor binding” and effect different from “Unspecified” and trust as “Present”, “Approved” or “Probably present (animal model)”). These 19 ligands interact with a subset of the CMPs through 31 interactions, taking into account that some ligands can bind several CMPs. All interactions between these nodes were selected, including the 31 previously described ligand→CMP interactions, resulting in 94 CMP→TF pathway-mediated interactions and 121 TF→node (CMP, TF or ligand) regulations. All the interactions were derived from MetaBase. The directionality of the CMP→TF interactions was determined by the number of inhibiting interactions: activating if the signaling steps from the CMP to the TF consisted of activating interactions or of an even number of inhibiting interactions; inhibitory, if the signaling steps from CMP to TF contained an uneven number of inhibitory interactions. When none of these conditions was met, the interaction was marked as unclear.

Interactions between a TF belonging to one of the 6 selected modules and a target node that is not a component of these modules were then filtered out. In addition, only interactions involving one of CSF1R’s first neighbor were kept in the network. Finally, nodes that were no longer connected were filtered out, leading to a network of 34 nodes, connected by 112 interactions (S1 Fig).

To reduce the number of variables in our model and thus avoid overfitting, the network was simplified as follows. 23 nodes could be removed because either they were a close form or a precursor of another node with which they shared the same set of interactions with the rest of the network (S2–S4 Figs), or because they were just intermediate of one or few interactions and could thus be replaced by the corresponding direct interaction(s) (S5 Fig). In addition, interactions with proteins were removed when they already existed at the level of the corresponding RNA. We also removed the remaining interactions with conflicting effect, i.e. that could be both activating or inhibiting depending on the pathway. This ended up with a network of 11 nodes (Fig 1A). Each transcriptional and post-translational regulation had been identified experimentally in others’ work (Table 1).

**Table 1:** Transcriptional and post-translational regulations of the gene regulatory network.

#	Regulations	Description	References
1	LPS ➔ STAT1	Activation of STAT1 protein by LPS	[18]
2	LPS ➔ NFκB	Activation of NFκB transcription by LPS	[19]
3	NFκB ➔ NFκB	Auto-activation of NFκB transcription	[20,21]
4	NFκB ➔ STAT1 NFκB ➔ IRF7 ➔ STAT1	Indirect activation of STAT1 transcription by NFκB: NFκB promotes transcription of IRF7 which, in its turn, induces STAT1 transcription	[22,23]
5	STAT1 ➔ STAT1	Auto-activation of STAT1 transcription	[24,25]
6	IRF8—STAT1	Inhibition of STAT1 transcription by IRF8	[26]
7	STAT1 ➔ IRF8	Activation of IRF8 transcription by STAT1	[27]
8	STAT1 ➔ IL6R	Activation of IL6R transcription by STAT1	[28]
9	IL6R ➔ STAT1	Activation of STAT1 protein by IL6R	[29]
10	NFκB ➔ PU1	Activation of PU1 transcription by NFKB	[30]
11	PU1 ➔ NFκB	Activation of NFκB transcription by PU1	[31]
12	C/EBPα ➔ NFκB	Activation of NFκB transcription by C/EBPα	[32]
13	TNFR1 ➔ NFκB	Activation of NFκB protein by TNFR1	[33]
14	C/EBPα ➔ IL6R	Activation of IL6R transcription by CEBPA	[34]
15	LPS—C/EBPα	Inhibition of C/EBPα transcription by LPS	[33]
16	C/EBPα ➔ TNFR1	Activation of TNFR1 transcription by C/EBPα	[35]
17	C/EBPα ➔ CSF1R	Activation of CSF1R transcription by C/EBPα	[33]
18	STAT3 ➔ TNFR1	Activation of TNFR1 transcription by STAT3	[33]
19	CSF1 ➔ CSF1R	Activation of CSF1R by CSF1	[36]
20	STAT1 ➔ CSF1	Activation of CSF1 transcription by STAT1	[37]
21	CSF1R ➔ STAT1	Activation of STAT1 protein by CSF1R	[26]
22	NFκB ➔ CSF1	Activation of CSF1 transcription by NFκB	[38]
23	STAT3 ➔ IL6R	Activation of IL6R transcription by STAT3	[39]
24	C/EBPα ➔ PU1	Activation of PU1 transcription by C/EBPα	[40]
25	PU1 ➔ C/EBPα	Activation of C/EBPα transcription by PU1	[32]
26	PU1 ➔ IRF8	Activation of IRF8 transcription by PU1	[41]
27	PU1 ➔ PU1	Auto-activation of PU1 transcription	[32]
28	PU1 ➔ CSF3R	Activation of CSF3R transcription by PU1	[42]
29	STAT1 ➔ CSF3R	Activation of CSF3R transcription by STAT1	[42]
30	STAT1 ➔ STAT3	Activation of STAT3 transcription by STAT1	[26]
31	STAT1 ➔ STAT3 STAT1 ➔ LIF & OSM receptors ➔ STAT3	Activation of STAT3 protein by STAT1: STAT1 promotes activation of LIF and OSM receptors which, in their turn, can promote activation of STAT3	[43,44]
32	STAT3 ➔ STAT1	Activation of STAT1 protein by STAT3	[26]
33	C/EBPα ➔ CSF3R	Activation of CSF3R transcription by C/EBPα	[36]
34	CSF3R ➔ CEBPA	Activation of C/EBPα protein by CSF3R	[45]
35	PU1 ➔ CSF1R	Activation of CSF1R transcription by PU1	[33]
36	PU1 ➔ CSF1	Activation of CSF1 transcription by PU1	[32]
37	STAT3 ➔ CSF1	Activation of CSF1 transcription by STAT3	[32]
38	STAT3 ➔ STAT3	Auto-activation of STAT3 transcription	[46]
39	CSF3R ➔ STAT3	Activation of STAT3 protein by CSF3R	[36]
40	STAT3 ➔ C/EBPα	Activation of C/EBPα transcription by STAT3	[47]
41	TNFR1 ➔ STAT3	Activation of STAT3 protein by TNFR1	[48]
42	CSF1R ➔ STAT3	Activation of STAT3 protein by CSF1R	[49]

### Mathematical model

The mathematical model is defined by a set of kinetic equations describing the temporal evolution of the mRNA and protein expression levels of *Csf1r*, *Csf1*, *Stat1*, *Stat3*, *Irf8*, *Nfkb*, *Pu1*, *Il6R*, *Csf3r*, *Tnfr1* and *Cebpa*. The mathematical model with the kinetic equations and the parameter values are described in S1 Text and Tables A-E in S1 Tables. Numerical simulations were performed with XPPAUTO (http://www.math.pitt.edu/~bard/xpp/xpp.html; [50]) and Matlab. The code is available in Github (https://github.com/ClaudeGerard/Gerard_et_al.Epilepsy)

### Cell culture experiments, LPS and siRNA treatments

BV-2 cell line were grown in DMEM (Lonza, Leusden, Netherlands) supplemented with fetal bovine serum (FBS) 10% (Merck, Darmstadt Germany), sodium pyruvate 1mM (Gibco, Waltham, MA, USA), penicillin-streptomycin 1% (Gibco) and amphotericin B 1% (Gibco). For LPS treatment, cells were seeded in 12 well plates and treated with 1 μg/ml of LPS for different time periods before protein or RNA extraction. For siRNA treatment, cells were seeded in 12 well plates and transfected with 50 nM siRNA (Dharmacon #D-001206-14-05, #M-058881-02-0005, #M-040794-00-0005, #M-040737-00-0005) for 4 h using Lipofectamine 2000 (Invitrogen #11668027). The medium was changed and cells were collected 24 h after siRNA targeting Irf8 or 48 h after siRNA administration targeting Stat1 and Stat3.

### RNA extraction and analysis

Total RNA was isolated from cultured BV-2 cells using Trizol (Invitrogen, Life technologies). cDNA was synthesized using MMLV reverse transcriptase (Invitrogen, Life technologies) according to manufacturer’s protocol. Gene expression was quantified by RT-qPCR using Kapa SYBR Fast 2X Universal Master Mix (Sopachem). mRNA levels were normalized to *β-actin* expression. *β-actin* Fwd: TCCTGAGCGCAAGTACTCTGT, *β-actin* Rev: CTGATCCACATCTGCTGGAAG; *Irf8* Fwd: CGTGGAAGACGAGGTTACGCTG, *Irf8* Rev: GCTGAATGGTGTGTGTCATAGGC; *Stat1* Fwd: CTGAATATTTCCCTCCTGGG, *Stat1* Rev: TCCCGTACAGATGTCCATGAT; *Stat3* Fwd: GGATCGCTGAGGTACAACCC, *Stat3* Rev: GTCAGGGGTCTCGACTGTCT; *Nfkb1* Fwd: TTCCGCTATGTGTGTGAAGG, *Nfkb1* Rev: GTCCTTGGGTCCTGCTGTTA; *Il6r* Fwd: AAGCAGCAGGCAATGTTACC, *Il6r* Rev: CATAAATAGTCCCCAGTGTCG; *Tnfr1* Fwd: CCGGGAGAAGAGGGATAGCTT, *Tnfr1* Rev: TCGGACAGTCACTCACCAAGT; *Csf1* Fwd: AGTATTGCCAAGGAGGTGTCAG, *Csf1* Rev: ATCTGGCATGAAGTCTCCATTT; *Csf1r* Fwd: GACCCTGAATCTCCCGGAAG, *Csf1r* Rev: GGTACAACGGTAGGTCCCAG; *Csf3r* Fwd: TGCACCCTGACTGGAGTTAC, *Csf3r* Rev: TGAAATCTCGATGTGTCCACAG; *Cebpa* Fwd:, *Cebpa* Rev:; *Pu1* Fwd: CACGTCAGAGGCAACGCTAA, *Pu1* Rev: ACCATTTGTTACACCTCTCCAGTCA. 2^-ΔCt^ method was used for gene quantification in Figs 1B and S6 and S7, while 2^-ΔΔCt^ method was used for gene quantification in Fig 3.

### Western blotting

Proteins were extracted with RIPA buffer (Tris-HCl 50 mM pH7.4, NaCl 150 mM, NP-40 1%, sodium deoxycholate 0.25%, sodium orthovanadate 1 mM) complemented with protease and protease/phosphatase inhibitors (Roche, Basel, Switzerland). Cell lysates were sonicated for 15 sec and centrifuged. Proteins were then measured using Bradford protein assay. 30 μg protein were loaded on SDS-polyacrylamide gels 7.5% and transferred onto a PVDF membrane (Merck). After transfer, membranes were blocked for 1 h in Tris-Buffered Saline (TBS) with Tween-20 0.1% (Sigma) and bovine serum albumin (BSA) 5% or milk 5% and then incubated overnight at 4°C with primary antibody (Table 2) in TBS-BSA 5% or milk 5%. Then, membranes were incubated with anti-rabbit (1:2000, Enzo Life Sciences, Plymouth Meeting, PA, USA) or anti-mouse (1:1000, Cell Signaling Technology, Danvers, Massachusetts, USA) secondary antibodies, and proteins were detected using chemiluminescence (Super Signal West Pico Plus, Thermo Fisher Scientific) and Fusion Solo S equipment (Vilber Lourmat, Collegien, France).

**Table 2:** Primary antibodies used in western blotting experiments.

Antibodies	Species	Dilution	Reference
STAT1	Rabbit	1/1000	Bioké #9172S
STAT3	Rabbit	1/1000	Bioké #12640S
IRF8	Goat	1/300	Abcam #ab28696
HSC70	Mouse IgG2a	1/5000	Bio-Connect #SC-7298

### Statistical analysis and normalization

We normalized the mRNA expression levels to the expression of *β-actin* mRNA (Figs 1B, S6 and S7). After a first normalization to *β-actin* mRNA, we normalized the mRNA expression to the control condition (Fig 4). Protein expression was first normalized to HSC70 and then normalized to the expression of the gene of interest in the control condition (Fig 4). Data are means ± SD. Significance was assessed by Student t-test.

## Supporting information

S1 Text Description and calibration of the mathematical model. (DOCX)

S1 Tables **Table A**. Variables of the mathematical model; **Table B**. Kinetic equations of the mathematical model; **Table C**. Parameters of the mathematical model; **Table D**. Initial conditions of the model; **Table E**. Half-life durations of the network components.
(DOCX)

S1 Fig 34-node network. Each arrow represents an interaction. The network has been divided in 3 sub-plots for clarity: (A) CMP→TF and ligand→CMP interactions; (B) TF→ligand and TF→CMP interactions; (C) TF→TF interactions. Interactions between CMP and TF occur via a signaling pathway. Interactions from a TF correspond to a transcriptional control. Interaction from ligand to CMP correspond to the activation or inhibition of the CMP by its ligand. Color code is indicated.
(PDF)

S2 Fig Integration of NF-κB in the network. NF-κB1 (p50) and NF-κB p50/p50 are precursors of NF-κB, and all upstream regulators of these precursors also regulate NF-κB. Therefore, the 2 precursors were removed. TNF-R1 activates NF-κB and has a conflicting effect on precursor NF-κB1 (p50): the well documented direct effect of NF-κB activation by TNF-R1 was kept.
(PDF)

S3 Fig Integration of IL6 receptor in the network. sIL6RA could be removed since sIL6RA is the soluble form of IL6RA, and both share the same interactions with the rest of the network. CNTF receptor is a component of gp130 and they both share the same interactions with the rest of the network; therefore CNTF receptor was removed. In addition, gp130 and IL6RA/sIL6RA are components of IL-6 receptor. All regulators of gp130 or IL6RA also regulate the IL-6 receptor. The network was thus simplified by removing IL6RA, sIL6RA and gp130. IL-6 receptor activates STAT1, while its components gp130/CNTF receptors have an unclear effect on the same TF. Again, the most direct regulation was kept, namely STAT1 activation by IL-6 receptor.
(PDF)

S4 Fig Integration of GM-CSF receptor in the network. CSF2RB is a subunit of GM-CSF receptor, and its interactions are covered by the latter. Therefore CSF2RB was filtered out. In addition, G-CSF and GM-CSF receptors are similar and share the same interactions within the network. The only exception is the additional activation of C/EBPα by G-CSF receptor (and not by GM-CSF receptor). Therefore, only G-CSF (CSF3R) receptor was maintained in the network.
(PDF)

S5 Fig Removal of simple nodes. (A) In each case presented in this figure, a CMP could be removed, while considering the sum of interactions between the remaining nodes. In this process, new unclear interactions are not kept in the network. Dashed lines, regulation of protein function; plain lines, transcriptional regulation. (B) Three sequential steps were followed after removal of CMPs. At each step, the TF could be removed, while considering the sum of interactions between the remaining nodes. (C) PTAFR can be removed without adding any interaction, as all the interactions it mediates are already present.
(PDF)

S6 Fig Experimental and *in silico* temporal dynamics of the GRN in the absence of LPS. Temporal evolution of the mRNA expression level of (A) *Tnfr1*, (B) *Pu1*, (C) *Stat3*, (D) *Nfkb*, (E) *Stat1*, (F) *Cebpa*, (G) *Csf3r*, (H) *Irf8*, (I) *Csf1*, (J) *Csf1r* and (K) *Il6r*. Red curves: experimental data, black curves: mathematical model. Experimental data are the means (relative to β-actin) +/- SD; n = 4 and are collected at the indicated time points in BV-2 cells (S5–S6 Data). Conditions of the numerical simulations are outlined in S1 Text and Tables A-E in S1 Tables.
(PDF)

S7 Fig Experimental and *in silico* temporal dynamics of the GRN in the presence of LPS. Temporal evolution of the mRNA expression levels of (A) *Tnfr1*, (B) *Pu1*, (C) *Nfkb*, (D) *Csf1r* and (E) *Csf3r*. LPS = 1 μM added at t = 0h. Red curves: experimental data, black curves: mathematical model. Experimental data are the means (relative to β-actin) +/- SD; n = 4 and are collected at the indicated time points after LPS administration in BV-2 cells (S7–S8 Data). Conditions of the numerical simulations are outlined in S1 Text and Tables A-E in S1 Tables.
(PDF)

S8 Fig *In silico* prediction of the impact of *Pu1*, *Csf3r*, *Il6r* and *Tnfr1* expression levels on the GRN dynamics. Impact of low (blue bars) or high expression levels (red bars) of (A) *Pu1*, (B) *Csf3r*, (C) *Il6r* and (D) *Tnfr1* on the mRNA expression levels of the other GRN components. The control conditions in the simulations (horizontal dashed lines) are the expression levels of the GRN components in the absence of LPS after 48h. Numerical simulations for the various conditions are described in S1 Text and Tables A-E in S1 Tables.
(PDF)

S9 Fig *In silico* prediction of the impact of *Nfkb*, *Csf1* and *Cebpa* expression levels on the GRN dynamics. Impact of low (blue bars) or high expression levels (red bars) of (A) *NfkB*, (B) *Csf1* and (C) *Cebpa* on the mRNA expression levels of the other GRN components. The control conditions in the simulations (horizontal dashed lines) are the expression levels of the GRN components in the absence of LPS after 48h. Numerical simulations for the various conditions are found in S1 Text and Tables A-E in S1 Tables.
(PDF)

S10 Fig *In silico* temporal dynamics of Stat1 mRNA in the absence or presence of LPS and with varying initial conditions. Temporal evolution of Stat1 mRNA in the absence (A) or presence (B) of LPS, using different initial conditions randomly chosen between the values of 0 and 20. Simulations were performed with XPPAUT. Parameter values are as in Table C in S1 Tables.
(PDF)

S1 Data Supporting data in.txt format. (TXT)

S2 Data Supporting data in.txt format. (TXT)

S3 Data Supporting data in.csv format. (CSV)

S4 Data Supporting data in.txt format. (TXT)

S5 Data Supporting data in.txt format. (TXT)

S6 Data Supporting data in.txt format. (TXT)

S7 Data Supporting data in.txt format. (TXT)

S8 Data Supporting data in.txt format. (TXT)
