# Supplementary material for: Temporal dynamics of a CSF1R signaling gene regulatory network involved in epilepsy
Source: PLoS Comput Biol. 2021 Apr 5;17(4):e1008854. doi: 10.1371/journal.pcbi.1008854 (PMC8057615; doi:10.1371/journal.pcbi.1008854)
Supplement: S1 Text — (DOCX) [file pcbi.1008854.s001.docx]

**S1. Text**

# **Description and calibration of the mathematical model**

The model is composed of 17 kinetic equations describing the temporal evolution of the expression level of each network component. The variables, parameters, initial conditions and numerical values used to calibrate the model are in Tables A-E in S1 Tables. Simulations were performed using XPPAUT (http://www.math.pitt.edu/~bard/xpp/xpp.html; [1]) and Matlab.

To fit the parameter values (Table C in S1 Tables), we considered the half-life of the mRNA of each GRN component, which allows us to determine the degradation rate constant of each mRNA. Then, we adjusted the value of the other parameters of the model to fit data in the time series.

Kinetic equations [1] to [11] describe the temporal evolution of the - *Irf8, Pu1, Csf3r, Il6r, Tnfr1, Csf1, Stat3, Csf1r, Cebpa, Nfkb and Stat1* mRNA levels. Kinetic equations [12] to [16] describe the time dynamics of the expression level of the active form of STAT3 (STAT3a), CSF1R (CSF1Ra), C/EBPα (CEBPAa), NFκB (NFKBa) and STAT1 (STAT1a) protein. Kinetic equation [17] represents the temporal evolution of LPS level, which was used in BV-2 cells to induce an inflammatory response.

Algebraic equations [18] to [22] describe the conservation relation between inactive and active form of the protein STAT3, CSF1R, C/EBPα, NFκB and STAT1, respectively. Algebraic equations [23] to [28] describe the total protein expression level of IRF8, PU1, CSF3R, IL6R, TNFR1, and CSF1. We consider post-translational regulations in the mathematical model that can activate or inhibit protein activity. Since STAT3, CSF1R, C/EBPα, NFκB and STAT1 are regulated post-translationally in the model (see scheme of the GRN in Fig. 1A), these proteins can switch between an active and an inactive state.

We consider that the total protein expression level of each component is 3000 higher (*cprot* = 3000) than the expression level of its corresponding mRNA. This protein *versus* mRNA expression ratio originates from Schwanhausser et al. [2], which shows that the mean protein/mRNA ratio measured over 5000 different genes is close to 3000.

Each component of the GRN in the model was quantitatively calibrated with the mRNA levels of each component measured by RT-qPCR in BV-2 cells. Experimentally, we normalize the mRNA expression level of each component to the expression of *β-actin*.

Note that the steady-state expression level of each component is given by the balance between their rates of synthesis and degradation. Half-life of mRNA for each component was based on the literature (Table E in S1 Tables).

We calibrated the mathematical model on the mRNA expression levels of each component of the GRN measured after 1h, 2h, 3h, 4h, 5h, 6h, 7h, 8h, 10h, 12h, 16h, 20h, 24, 28h and 32h after LPS treatment (1 μM). For the control condition, the same measurements were performed at the same time points in the absence of LPS treatment.

To account for the temporal evolution of the mRNA expression levels in the presence of LPS, we had to consider that LPS activates the transcription of each network component (see parameter *LPS2* in the equations). In addition, to determine the bifurcation diagrams in Figs 4 and 5 as a function of LPS, we replaced LPS2 by LPS in the kinetic equations of the model.

# ***In silico* downregulation and overexpression of the network components**

In the following conditions, LPS = 0 and the other parameter values, if not indicated, are as in Table C in S1 Tables.

Modification of *Stat1* expression (condition in Fig 2A):

- Stat1 downregulation: *V*_M1STAT1_ = 0.00005, *V*_M2STAT1_ = *V*_M3STAT1_ = 0.0001.
- Stat1 overexpression: *V*_M1STAT1_ = 0.0001, *V*_M2STAT1_ = *V*_M3STAT1_ = 0.015.

Modification of *Stat3* expression (condition in Fig 2B):

- Stat3 downregulation: *V*_M1STAT3_ = *V*_M2STAT3_ = 0.00001, *V*_M3STAT3_ = 0.00021.
- Stat3 overexpression: *V*_M1STAT3_ = *V*_M2STAT3_ = 0.00001, *V*_M3STAT3_ = 0.021.

Modification of *Irf8* expression (condition in Fig 2C):

- Irf8 downregulation: *V*_M1IRF8_ = 0.0000014, *V*_M2IRF8_ = 0.0003, *V*_M3IRF8_ = 0.0062.
- Irf8 overexpression: *V*_M1IRF8_ = 0.0000014, *V*_M2IRF8_ = 0.03, *V*_M3IRF8_ = 0.62.

Modification of *Csf1r* expression (condition in Fig 2D):

- Csf1r downregulation: *V*_M1CSF1R_ = 0.000001, *V*_M2CSF1R_ = 0.00001, *V*_M3CSF1R_ = 0.0004, *V*_M4CSF1R_ = 0.000005.
- Csf1r overexpression: *V*_M1CSF1R_ = 0.0001, *V*_M2CSF1R_ = 0.001, *V*_M3CSF1R_ = 0.04, *V*_M4CSF1R_ = 0.00005.

Inhibition of *Stat1* expression (condition in Fig 3A):

- Simulation of Stat1 siRNA treatment: *V*_M2STAT1_ = *V*_M3STAT1_ = 0.00045.

Inhibition of Stat3 expression (condition in Fig 3B):

- Simulation of Stat3 siRNA treatment: *V*_M3STAT3_ = 0.0011.

Inhibition of *Irf8* expression (condition in Fig 3B):

- Simulation of Irf8 siRNA treatment: *V*_M2IRF8_ = 0.0015, *V*_M3IRF8_ = 0.03.

Modification of *Pu1* expression (condition in S8A Fig):

- Pu1 downregulation: *V*_M1PU1_ = 0.0000005, *V*_M2PU1_ = 0.00008, *V*_M3PU1_ = 0.00025, *V*_M4PU1_ = 0.00006.
- Pu1 overexpression: *V*_M1PU1_ = 0.000005, *V*_M2PU1_ = 0.008, *V*_M3PU1_ = 0.015, *V*_M4PU1_ = 0.006.

Modification of Csf3r expression (condition in S8B Fig):

- Csf3r downregulation: *V*_M1CSF3R_ = 0.000006, *V*_M2CSF3R_ = 0.0001, *V*_M3CSF3R_ = 0.005, *V*_M4CSF3R_ = 0.0006, *V*_M5CSF3R_ = 0.00001.
- Csf3r overexpression: *V*_M1CSF3R_ = 0.00000006, *V*_M2CSF3R_ = 0.000001, *V*_M3CSF3R_ = 0.00005, *V*_M4CSF3R_ = 0.000006, *V*_M5CSF3R_ = 0.0000001.

Modification of *Il6r* expression (condition in S8C Fig):

- Il6r downregulation: *V*_M1IL6R_ = 0.0000005, *V*_M2IL6R_ = 0.00006, *V*_M3IL6R_ = 0.000001, *V*_M4IL6R_ = 0.00001.
- Il6r overexpression: *V*_M1IL6R_ = 0.0000005, *V*_M2IL6R_ = 0.005, *V*_M3IL6R_ = 0.000001, *V*_M4IL6R_ = 0.001.

Modification of *Tnfr1* expression (condition in S8D Fig):

- Tnfr1 downregulation: *V*_M1TNFR1_ = 0.0000002, *V*_M2TNFR1_ = 0.000005, *V*_M3TNFR1_ = 0.001.
- Tnfr1 overexpression: *V*_M1TNFR1_ = 0.00002, *V*_M2TNFR1_ = 0.0005, *V*_M3TNFR1_ = 0.1.

Modification of *Nfkb* expression (condition in S9A Fig):

- Nfkb downregulation: *V*_M1NFKB_ = 0.00000067, *V*_M2NFKB_ = 0.00018, *V*_M3NFKB_ = 0.0018, *V*_M4NFKB_ = 0.00006, *V*_M5NFKB_ = 0.0002.
- Nfkb overexpression: *V*_M1NFKB_ = 0.00000067, *V*_M2NFKB_ = 0.018, *V*_M3NFKB_ = 0.18, *V*_M4NFKB_ = 0.006, *V*_M5NFKB_ = 0.002.

Modification of *Csf1* expression (condition in S9B Fig):

- Csf1 downregulation: *V*_M1CSF1_ = 0.000005, *V*_M2CSF1_ = 0.0002, *V*_M3CSF1_ = 0.019, *V*_M4CSF1_ = 0.0007, *V*_M5CSF1_ = 0.002.
- Csf1 overexpression: *V*_M1CSF1_ = 0.00005, *V*_M2CSF1_ = 0.02, *V*_M3CSF1_ = 1.5, *V*_M4CSF1_ = 0.07, *V*_M5CSF1_ = 0.2.

Modification of *Cebpa* expression (condition in S9C Fig):

- Cebpa downregulation: *V*_M1CEBPA_ = 0.00008, *V*_M2CEBPA_ = 0.00005, *V*_M3CEBPA_ = 0.00008.
- Cebpa overexpression: *V*_M1CEBPA_ = 0.0001, *V*_M2CEBPA_ = 0.005, *V*_M3CEBPA_ = 0.008.

**References**

1. Ermentrout B. Simulating, analyzing, and animating dynamical systems: a guide to XPPAUT for researchers and students. Philadelphia: Society for Industrial and Applied Mathematics; 2002.

2. Schwanhausser B, Busse D, Li N, Dittmar G, Schuchhardt J, Wolf J, et al. Global quantification of mammalian gene expression control. Nature. 2011;473(7347):337-42. doi: 10.1038/nature10098. PubMed PMID: 21593866.
