# Supplementary material for: Temporal dynamics of a CSF1R signaling gene regulatory network involved in epilepsy
Source: PLoS Comput Biol. 2021 Apr 5;17(4):e1008854. doi: 10.1371/journal.pcbi.1008854 (PMC8057615; doi:10.1371/journal.pcbi.1008854)
Supplement: S1 Tables — Table A. Variables of the mathematical model; Table B. Kinetic equations of the mathematical model; Table C. Parameters of the mathematical model; Table D. Initial conditions of the model; Table E. Half-life durations of the network components. (DOCX) [file pcbi.1008854.s002.docx]

# **S1. Supporting Tables**

**S1 Table A. Variables of the mathematical model.**

| **Name** | **Equation** | **Definition** |
| --- | --- | --- |
| *mIRF8* | 1 | IRF8 mRNA |
| *mPU1* | 2 | PU1 mRNA |
| *mCSF3R* | 3 | CSF3R mRNA |
| *mIL6R* | 4 | IL6R mRNA |
| *mTNFR1* | 5 | TNFR1 mRNA |
| *mCSF1* | 6 | CSF1 mRNA |
| *mSTAT3* | 7 | STAT3 mRNA |
| *mCSF1R* | 8 | CSF1R mRNA |
| *mCEBPA* | 9 | CEBPA mRNA |
| *mNFKB* | 10 | NFKB mRNA |
| *mSTAT1* | 11 | STAT1 mRNA |
| *STAT3a* | 12 | Active form of STAT3 |
| *CSF1Ra* | 13 | Active form of CSF1R |
| *CEBPAa* | 14 | Active form of CEBPA |
| *NFKBa* | 15 | Active form of NFKB |
| *STAT1a* | 16 | Active form of STAT1 |
| *LPS* | 17 | Level of LPS |
| *STAT3i* | 18 | Inactive form of STAT3 |
| *CSF1Ri* | 19 | Inactive form of CSF1R |
| *CEBPAi* | 20 | Inactive form of CEBPA |
| *NFKBi* | 21 | Inactive form of NFKB |
| *STAT1i* | 22 | Inactive form of STAT1 |
| *IRF8* | 23 | Total form of IRF8 |
| *PU1* | 24 | Total form of PU1 |
| *CSF3R* | 25 | Total form of CSF3R |
| *IL6R* | 26 | Total form of IL6R |
| *TNFR1* | 27 | Total form of TNFR1 |
| *CSF1* | 28 | Total form of CSF1 |

**S1 Table B. Kinetic equations of the mathematical model.**

| $\frac{\boldsymbol{dmIRF}\boldsymbol{8}}{\boldsymbol{dt}}\boldsymbol{=}\boldsymbol{V}_{\mathbf{M1IRF8}}\boldsymbol{+}\boldsymbol{V}_{\mathbf{M2IRF8}}\boldsymbol{\cdot}\left( \frac{\boldsymbol{PU}\boldsymbol{1}}{\boldsymbol{K}_{\mathbf{AS1PU1}}\boldsymbol{+PU}\boldsymbol{1}} \right)\boldsymbol{+}\boldsymbol{V}_{\mathbf{M3IRF8}}\boldsymbol{\cdot}\left( \frac{{\boldsymbol{STAT}\boldsymbol{1}\boldsymbol{a}}^{\boldsymbol{n}}}{{\boldsymbol{K}_{\mathbf{STAT1A}}}^{\boldsymbol{n}}\boldsymbol{+}{\boldsymbol{STAT}\boldsymbol{1}\boldsymbol{a}}^{\boldsymbol{n}}} \right)\boldsymbol{+LPS}\boldsymbol{2\cdot LPSirf}\boldsymbol{8-}\boldsymbol{k}_{\boldsymbol{DMIRF}\boldsymbol{8}}\boldsymbol{\cdot mIRF}\boldsymbol{8}$ **[1]**  $\frac{\boldsymbol{dmPU}\boldsymbol{1}}{\boldsymbol{dt}}\boldsymbol{=}\boldsymbol{V}_{\mathbf{M1PU1}}\boldsymbol{+}\boldsymbol{V}_{\mathbf{M2PU1}}\boldsymbol{\cdot}\left( \frac{\boldsymbol{PU}\boldsymbol{1}}{\boldsymbol{K}_{\mathbf{AS2PU1}}\boldsymbol{+PU}\boldsymbol{1}} \right)\boldsymbol{+}\boldsymbol{V}_{\mathbf{M3PU1}}\boldsymbol{\cdot}\left( \frac{\boldsymbol{CEBPAa}^{\boldsymbol{n}}}{{\boldsymbol{K}_{\mathbf{AS1CEBPA}}}^{\boldsymbol{n}}\boldsymbol{+}\boldsymbol{CEBPAa}^{\boldsymbol{n}}} \right)\boldsymbol{+}\boldsymbol{V}_{\mathbf{M4PU1}}\boldsymbol{\cdot}\left( \frac{\boldsymbol{NFKBa}^{\boldsymbol{n}}}{{\boldsymbol{K}_{\mathbf{S5ANFKB}}}^{\boldsymbol{n}}\boldsymbol{+}\boldsymbol{NFKBa}^{\boldsymbol{n}}} \right)\boldsymbol{+LPS}\boldsymbol{2\cdot LPSpu}\boldsymbol{1-}\boldsymbol{k}_{\boldsymbol{DMPU}\boldsymbol{1}}\boldsymbol{\cdot mPU}\boldsymbol{1}$ **[2]**  $\frac{\boldsymbol{dmCSF}\boldsymbol{3}\boldsymbol{R}}{\boldsymbol{dt}}\boldsymbol{=}\boldsymbol{V}_{\mathbf{M1CSF3R}}\boldsymbol{+}\boldsymbol{V}_{\mathbf{M2CSF3R}}\boldsymbol{\cdot}\left( \frac{\boldsymbol{PU}\boldsymbol{1}}{\boldsymbol{K}_{\mathbf{AS3PU1}}\boldsymbol{+PU}\boldsymbol{1}} \right)\boldsymbol{+}\boldsymbol{V}_{\mathbf{M3CSF3R}}\boldsymbol{\cdot}\left( \frac{\boldsymbol{CEBPAa}^{\boldsymbol{n}}}{{\boldsymbol{K}_{\mathbf{AS2CEBPA}}}^{\boldsymbol{n}}\boldsymbol{+}\boldsymbol{CEBPAa}^{\boldsymbol{n}}} \right)\boldsymbol{+}\boldsymbol{V}_{\mathbf{M4CSF3R}}\boldsymbol{\cdot}\left( \frac{{\boldsymbol{STAT}\boldsymbol{3}\boldsymbol{a}}^{\boldsymbol{n}}}{{\boldsymbol{K}_{\mathbf{AS4STAT3A}}}^{\boldsymbol{n}}\boldsymbol{+}{\boldsymbol{STAT}\boldsymbol{3}\boldsymbol{a}}^{\boldsymbol{n}}} \right)\boldsymbol{+}\boldsymbol{V}_{\mathbf{M5CSF3R}}\boldsymbol{\cdot}\left( \frac{{\boldsymbol{STAT}\boldsymbol{1}\boldsymbol{a}}^{\boldsymbol{n}}}{{\boldsymbol{K}_{\mathbf{STAT1A5}}}^{\boldsymbol{n}}\boldsymbol{+}{\boldsymbol{STAT}\boldsymbol{1}\boldsymbol{a}}^{\boldsymbol{n}}} \right)\boldsymbol{+LPS}\boldsymbol{2\cdot LPScsf}\boldsymbol{3}\boldsymbol{r-}\boldsymbol{k}_{\boldsymbol{DMCSF}\boldsymbol{3}\boldsymbol{R}}\boldsymbol{\cdot mCSF}\boldsymbol{3}\boldsymbol{R}$ **[3]**  $\frac{\boldsymbol{dmIL}\boldsymbol{6}\boldsymbol{R}}{\boldsymbol{dt}}\boldsymbol{=}\boldsymbol{V}_{\mathbf{M1IL6R}}\boldsymbol{+}\boldsymbol{V}_{\mathbf{M2IL6R}}\boldsymbol{\cdot}\left( \frac{\boldsymbol{CEBPAa}^{\boldsymbol{n}}}{{\boldsymbol{K}_{\mathbf{AS3CEBPA}}}^{\boldsymbol{n}}\boldsymbol{+}\boldsymbol{CEBPAa}^{\boldsymbol{n}}} \right)\boldsymbol{+}\boldsymbol{V}_{\mathbf{M3IL6R}}\boldsymbol{\cdot}\left( \frac{{\boldsymbol{STAT}\boldsymbol{3}\boldsymbol{a}}^{\boldsymbol{n}}}{{\boldsymbol{K}_{\mathbf{AS5CEBPA}}}^{\boldsymbol{n}}\boldsymbol{+}{\boldsymbol{STAT}\boldsymbol{3}\boldsymbol{a}}^{\boldsymbol{n}}} \right)\boldsymbol{+}\boldsymbol{V}_{\mathbf{M4IL6R}}\boldsymbol{\cdot}\left( \frac{{\boldsymbol{STAT}\boldsymbol{1}\boldsymbol{a}}^{\boldsymbol{n}}}{{\boldsymbol{K}_{\mathbf{AS6CEBPA}}}^{\boldsymbol{n}}\boldsymbol{+}{\boldsymbol{STAT}\boldsymbol{1}\boldsymbol{a}}^{\boldsymbol{n}}} \right)\boldsymbol{+LPS}\boldsymbol{2\cdot LPSil}\boldsymbol{6}\boldsymbol{r-}\boldsymbol{k}_{\boldsymbol{DMIL}\boldsymbol{6}\boldsymbol{R}}\boldsymbol{\cdot mIL}\boldsymbol{6}\boldsymbol{R}$ **[4]**  $\frac{\boldsymbol{dmTNFR}\boldsymbol{1}}{\boldsymbol{dt}}\boldsymbol{=}\boldsymbol{V}_{\mathbf{M1TNFR1}}\boldsymbol{+}\boldsymbol{V}_{\mathbf{M2TNFR1}}\boldsymbol{\cdot}\left( \frac{{\boldsymbol{STAT}\boldsymbol{3}\boldsymbol{a}}^{\boldsymbol{n}}}{{\boldsymbol{K}_{\mathbf{ASSTAT3A}}}^{\boldsymbol{n}}\boldsymbol{+}{\boldsymbol{STAT}\boldsymbol{3}\boldsymbol{a}}^{\boldsymbol{n}}} \right)\boldsymbol{+}\boldsymbol{V}_{\mathbf{M3TNFR1}}\boldsymbol{\cdot}\left( \frac{\boldsymbol{CEBPA}^{\boldsymbol{n}}}{{\boldsymbol{K}_{\mathbf{AS7CEBPA}}}^{\boldsymbol{n}}\boldsymbol{+}\boldsymbol{CEBPA}^{\boldsymbol{n}}} \right)\boldsymbol{+LPS}\boldsymbol{2\cdot LPStnfr}\boldsymbol{1-}\boldsymbol{k}_{\boldsymbol{DMTNFR}\boldsymbol{1}}\boldsymbol{\cdot mTNFR}\boldsymbol{1}$ **[5]**  $\frac{\boldsymbol{dmCSF}\boldsymbol{1}}{\boldsymbol{dt}}\boldsymbol{=}\boldsymbol{V}_{\mathbf{M1CSF1}}\boldsymbol{+}\boldsymbol{V}_{\mathbf{M2CSF1}}\boldsymbol{\cdot}\left( \frac{\boldsymbol{NFKBa}^{\boldsymbol{n}}}{{\boldsymbol{K}_{\mathbf{S1ANFKB}}}^{\boldsymbol{n}}\boldsymbol{+}\boldsymbol{NFKBa}^{\boldsymbol{n}}} \right)\boldsymbol{+}\boldsymbol{V}_{\mathbf{M3CSF1}}\boldsymbol{\cdot}\left( \frac{{\boldsymbol{STAT}\boldsymbol{1}\boldsymbol{a}}^{\boldsymbol{n}}}{{\boldsymbol{K}_{\mathbf{STAT1A2}}}^{\boldsymbol{n}}\boldsymbol{+}{\boldsymbol{STAT}\boldsymbol{1}\boldsymbol{a}}^{\boldsymbol{n}}} \right)\boldsymbol{+}\boldsymbol{V}_{\mathbf{M4CSF1}}\boldsymbol{\cdot}\left( \frac{{\boldsymbol{STAT}\boldsymbol{3}\boldsymbol{a}}^{\boldsymbol{n}}}{{\boldsymbol{K}_{\mathbf{AS2STAT3A}}}^{\boldsymbol{n}}\boldsymbol{+}{\boldsymbol{STAT}\boldsymbol{3}\boldsymbol{a}}^{\boldsymbol{n}}} \right)\boldsymbol{+}\boldsymbol{V}_{\mathbf{M5CSF1}}\boldsymbol{\cdot}\left( \frac{\boldsymbol{PU}\boldsymbol{1}}{\boldsymbol{K}_{\mathbf{AS5PU1}}\boldsymbol{+PU}\boldsymbol{1}} \right)\boldsymbol{+LPS}\boldsymbol{2\cdot LPScsf}\boldsymbol{1-}\boldsymbol{k}_{\boldsymbol{DMCSF}\boldsymbol{1}}\boldsymbol{\cdot mCSF}\boldsymbol{1}$ **[6]**  $\frac{\boldsymbol{dmSTAT}\boldsymbol{3}}{\boldsymbol{dt}}\boldsymbol{=}\boldsymbol{V}_{\mathbf{M1STAT3}}\boldsymbol{+}\boldsymbol{V}_{\mathbf{M2STAT3}}\boldsymbol{\cdot}\left( \frac{{\boldsymbol{STAT}\boldsymbol{3}\boldsymbol{a}}^{\boldsymbol{n}}}{{\boldsymbol{K}_{\mathbf{STAT3A}}}^{\boldsymbol{n}}\boldsymbol{+}{\boldsymbol{STAT}\boldsymbol{3}\boldsymbol{a}}^{\boldsymbol{n}}} \right)\boldsymbol{+}\boldsymbol{V}_{\mathbf{M3STAT3}}\boldsymbol{\cdot}\left( \frac{{\boldsymbol{STAT}\boldsymbol{1}\boldsymbol{a}}^{\boldsymbol{n}}}{{\boldsymbol{K}_{\mathbf{STAT1A4}}}^{\boldsymbol{n}}\boldsymbol{+}{\boldsymbol{STAT}\boldsymbol{1}\boldsymbol{a}}^{\boldsymbol{n}}} \right)\boldsymbol{+LPS}\boldsymbol{2\cdot LPSstat}\boldsymbol{3-}\boldsymbol{k}_{\boldsymbol{DMSTAT}\boldsymbol{3}}\boldsymbol{\cdot mSTAT}\boldsymbol{3}$ **[7]**  $\frac{\boldsymbol{dmCSF}\boldsymbol{1}\boldsymbol{R}}{\boldsymbol{dt}}\boldsymbol{=}\boldsymbol{V}_{\mathbf{M1CSF1R}}\boldsymbol{+}\boldsymbol{V}_{\mathbf{M2CSF1R}}\boldsymbol{\cdot}\left( \frac{\boldsymbol{PU}\boldsymbol{1}}{\boldsymbol{K}_{\mathbf{AS4PU1}}\boldsymbol{+PU}\boldsymbol{1}} \right)\boldsymbol{+}\boldsymbol{V}_{\mathbf{M3CSF1R}}\boldsymbol{\cdot}\left( \frac{\boldsymbol{CEBPAa}^{\boldsymbol{n}}}{{\boldsymbol{K}_{\mathbf{AS4CEBPA}}}^{\boldsymbol{n}}\boldsymbol{+}\boldsymbol{CEBPAa}^{\boldsymbol{n}}} \right)\boldsymbol{+}\boldsymbol{V}_{\mathbf{M4CSF1R}}\boldsymbol{\cdot}\left( \frac{{\boldsymbol{STAT}\boldsymbol{3}\boldsymbol{a}}^{\boldsymbol{n}}}{{\boldsymbol{K}_{\mathbf{AS5STAT3A}}}^{\boldsymbol{n}}\boldsymbol{+}{\boldsymbol{STAT}\boldsymbol{3}\boldsymbol{a}}^{\boldsymbol{n}}} \right)\boldsymbol{+LPS}\boldsymbol{2\cdot LPSmcsf}\boldsymbol{1}\boldsymbol{r-}\boldsymbol{k}_{\boldsymbol{DMCSF}\boldsymbol{1}\boldsymbol{R}}\boldsymbol{\cdot mCSF}\boldsymbol{1}\boldsymbol{R}$ **[8]**  $\frac{\boldsymbol{dmCEBPA}}{\boldsymbol{dt}}\boldsymbol{=}\boldsymbol{V}_{\mathbf{M1CEBPA}}\boldsymbol{+}\left( \boldsymbol{V}_{\mathbf{M2CEBPA}}\boldsymbol{\cdot}\left( \frac{\boldsymbol{PU}\boldsymbol{1}}{\boldsymbol{K}_{\mathbf{AS10PU1}}\boldsymbol{+PU}\boldsymbol{1}} \right)\boldsymbol{+}\boldsymbol{V}_{\mathbf{M3CEBPA}}\boldsymbol{\cdot}\left( \frac{{\boldsymbol{STAT}\boldsymbol{3}\boldsymbol{a}}^{\boldsymbol{n}}}{{\boldsymbol{K}_{\mathbf{AS3STAT3A}}}^{\boldsymbol{n}}\boldsymbol{+}{\boldsymbol{STAT}\boldsymbol{3}\boldsymbol{a}}^{\boldsymbol{n}}} \right) \right)\boldsymbol{\cdot}\left( \frac{\boldsymbol{K}_{\boldsymbol{2}\boldsymbol{LPS}}}{\boldsymbol{K}_{\boldsymbol{2}\boldsymbol{LPS}}\boldsymbol{+LPS}} \right)\boldsymbol{-}\boldsymbol{k}_{\boldsymbol{DMCEBPA}}\boldsymbol{\cdot mCEBPA}$ **[9]**  $\frac{\boldsymbol{dmNFKB}}{\boldsymbol{dt}}\boldsymbol{=}\boldsymbol{V}_{\mathbf{M1NFKB}}\boldsymbol{+}\boldsymbol{V}_{\mathbf{M2NFKB}}\boldsymbol{\cdot}\left( \frac{\boldsymbol{NFKBa}^{\boldsymbol{n}}}{{\boldsymbol{K}_{\mathbf{S3ANFKB}}}^{\boldsymbol{n}}\boldsymbol{+}\boldsymbol{NFKBa}^{\boldsymbol{n}}} \right)\boldsymbol{+}\boldsymbol{V}_{\mathbf{M3NFKB}}\boldsymbol{\cdot}\left( \frac{\boldsymbol{LPS}}{\boldsymbol{K}_{\boldsymbol{LPS}}\boldsymbol{+LPS}} \right)\boldsymbol{+}\boldsymbol{V}_{\mathbf{M4NFKB}}\boldsymbol{\cdot}\left( \frac{\boldsymbol{PU}\boldsymbol{1}}{\boldsymbol{K}_{\boldsymbol{AS}\boldsymbol{6}\boldsymbol{PU}\boldsymbol{1}}\boldsymbol{+PU}\boldsymbol{1}} \right)\boldsymbol{+}\boldsymbol{V}_{\mathbf{M5NFKB}}\boldsymbol{\cdot}\left( \frac{\boldsymbol{CEBPAa}^{\boldsymbol{n}}}{{\boldsymbol{K}_{\boldsymbol{AS}\boldsymbol{8}\boldsymbol{CEBPA}}}^{\boldsymbol{n}}\boldsymbol{+}\boldsymbol{CEBPAa}^{\boldsymbol{n}}} \right)\boldsymbol{-}\boldsymbol{k}_{\boldsymbol{DMNFKB}}\boldsymbol{\cdot mNFKB}$ **[10]**  $\frac{\boldsymbol{dmSTAT}\boldsymbol{1}}{\boldsymbol{dt}}\boldsymbol{=}\boldsymbol{V}_{\mathbf{M1STAT1}}\boldsymbol{+}\left( \boldsymbol{V}_{\mathbf{M2STAT1}}\boldsymbol{\cdot}\left( \frac{{\boldsymbol{STAT}\boldsymbol{1}\boldsymbol{a}}^{\boldsymbol{n}}}{{\boldsymbol{K}_{\mathbf{SSTAT1A3}}}^{\boldsymbol{n}}\boldsymbol{+}{\boldsymbol{STAT}\boldsymbol{1}\boldsymbol{a}}^{\boldsymbol{n}}} \right)\boldsymbol{+}\boldsymbol{V}_{\mathbf{M3STAT1}}\boldsymbol{\cdot}\left( \frac{\boldsymbol{NFKBa}^{\boldsymbol{n}}}{{\boldsymbol{K}_{\mathbf{S4ANFKB}}}^{\boldsymbol{n}}\boldsymbol{+}\boldsymbol{NFKBa}^{\boldsymbol{n}}} \right)\boldsymbol{+LPS}\boldsymbol{2\cdot LPSstat}\boldsymbol{1} \right)\boldsymbol{\cdot}\left( \frac{\boldsymbol{K}_{\boldsymbol{IIRF}\boldsymbol{8}}}{\boldsymbol{K}_{\boldsymbol{IRF}\boldsymbol{8}}\boldsymbol{+IRF}\boldsymbol{8}} \right)\boldsymbol{-}\boldsymbol{k}_{\boldsymbol{DMSTAT}\boldsymbol{1}}\boldsymbol{\cdot mSTAT}\boldsymbol{1}$ **[11]**  $\frac{\boldsymbol{dSTAT}\boldsymbol{3}\boldsymbol{a}}{\boldsymbol{dt}}\boldsymbol{=}\left( \boldsymbol{V}_{\mathbf{A1STAT3}}\boldsymbol{\cdot TNFR}\boldsymbol{1+}\boldsymbol{V}_{\mathbf{A2STAT3}}\boldsymbol{\cdot STAT}\boldsymbol{1}\boldsymbol{a+}\boldsymbol{V}_{\mathbf{A3STAT3}}\boldsymbol{\cdot CSF}\boldsymbol{3}\boldsymbol{R+}\boldsymbol{V}_{\mathbf{A4STAT3}}\boldsymbol{\cdot CSF}\boldsymbol{1}\boldsymbol{Ra} \right)\boldsymbol{\cdot}\left( \frac{\boldsymbol{STAT}\boldsymbol{3}\boldsymbol{i}}{\boldsymbol{K}_{\boldsymbol{ASTAT}\boldsymbol{3}}\boldsymbol{+STAT}\boldsymbol{3}\boldsymbol{i}} \right)\boldsymbol{-}\boldsymbol{V}_{\boldsymbol{ISTAT}\boldsymbol{3}}\boldsymbol{\cdot}\left( \frac{\boldsymbol{STAT}\boldsymbol{3}\boldsymbol{a}}{\boldsymbol{K}_{\boldsymbol{ISTAT}\boldsymbol{3}}\boldsymbol{+STAT}\boldsymbol{3}\boldsymbol{a}} \right)$ **[12]**  $\frac{\boldsymbol{dCSF}\boldsymbol{1Ra}}{\boldsymbol{dt}}\boldsymbol{=}\boldsymbol{V}_{\mathbf{ACSF1R}}\boldsymbol{\cdot CSF}\boldsymbol{1\cdot}\left( \frac{\boldsymbol{CSF}\boldsymbol{1}\boldsymbol{Ri}}{\boldsymbol{K}_{\boldsymbol{ACSF}\boldsymbol{1}\boldsymbol{R}}\boldsymbol{+CSF}\boldsymbol{1}\boldsymbol{Ri}} \right)\boldsymbol{-}\boldsymbol{V}_{\boldsymbol{ICSF}\boldsymbol{1}\boldsymbol{R}}\boldsymbol{\cdot}\left( \frac{\boldsymbol{CSF}\boldsymbol{1}\boldsymbol{Ra}}{\boldsymbol{K}_{\boldsymbol{ICSF}\boldsymbol{1}\boldsymbol{R}}\boldsymbol{+CSF}\boldsymbol{1}\boldsymbol{Ra}} \right)$ **[13]**  $\frac{\boldsymbol{dCEBPAa}}{\boldsymbol{dt}}\boldsymbol{=}\boldsymbol{V}_{\mathbf{ACEBPA}}\boldsymbol{\cdot CSF}\boldsymbol{3}\boldsymbol{R\cdot}\left( \frac{\boldsymbol{CEBPAi}}{\boldsymbol{K}_{\boldsymbol{ACEBPA}}\boldsymbol{+CEBPAi}} \right)\boldsymbol{-}\boldsymbol{V}_{\boldsymbol{ICEBPA}}\boldsymbol{\cdot}\left( \frac{\boldsymbol{CEBPAa}}{\boldsymbol{K}_{\boldsymbol{ICEBPA}}\boldsymbol{+CEBPAa}} \right)$ **[14]**  $\frac{\boldsymbol{dNFKBa}}{\boldsymbol{dt}}\boldsymbol{=}\boldsymbol{V}_{\mathbf{ANFKB}}\boldsymbol{\cdot TNFR}\boldsymbol{1\cdot}\left( \frac{\boldsymbol{NFKBi}}{\boldsymbol{K}_{\boldsymbol{ANFKB}}\boldsymbol{+NFKBi}} \right)\boldsymbol{-}\boldsymbol{V}_{\boldsymbol{INFKB}}\boldsymbol{\cdot}\left( \frac{\boldsymbol{NFKBa}}{\boldsymbol{K}_{\boldsymbol{INFKB}}\boldsymbol{+NFKBa}} \right)$ **[15]**  $\frac{\boldsymbol{dSTAT}\boldsymbol{1}\boldsymbol{a}}{\boldsymbol{dt}}\boldsymbol{=}\left( \boldsymbol{V}_{\mathbf{A1STAT1}}\boldsymbol{\cdot IL}\boldsymbol{6}\boldsymbol{R+}\boldsymbol{V}_{\mathbf{A2STAT1}}\boldsymbol{\cdot CSF}\boldsymbol{1}\boldsymbol{Ra+}\boldsymbol{V}_{\mathbf{A3STAT1}}\boldsymbol{\cdot STAT}\boldsymbol{3}\boldsymbol{a+}\boldsymbol{V}_{\mathbf{A4STAT1}}\boldsymbol{\cdot LPS} \right)\boldsymbol{\cdot}\left( \frac{\boldsymbol{STAT}\boldsymbol{1}\boldsymbol{i}}{\boldsymbol{K}_{\boldsymbol{ASTAT}\boldsymbol{1}}\boldsymbol{+STAT}\boldsymbol{1}\boldsymbol{i}} \right)\boldsymbol{-}\boldsymbol{V}_{\boldsymbol{ISTAT}\boldsymbol{1}}\boldsymbol{\cdot}\left( \frac{\boldsymbol{STAT}\boldsymbol{1}\boldsymbol{a}}{\boldsymbol{K}_{\boldsymbol{ISTAT}\boldsymbol{1}}\boldsymbol{+STAT}\boldsymbol{1}\boldsymbol{a}} \right)$ **[16]**  $\frac{\boldsymbol{dLPS}}{\boldsymbol{dt}}\boldsymbol{=}\boldsymbol{V}_{\mathbf{SLPS}}\boldsymbol{-}\boldsymbol{k}_{\mathbf{DLPS}}\boldsymbol{\cdot LPS}$ **[17]**  $\boldsymbol{STAT}\boldsymbol{3i=cPROT\cdot mSTAT}\boldsymbol{3-STAT}\boldsymbol{3}\boldsymbol{a}$ **[18]**  $\boldsymbol{CSF}\boldsymbol{1}\boldsymbol{Ri=cPROT\cdot mCSF}\boldsymbol{1}\boldsymbol{R-CSF}\boldsymbol{1}\boldsymbol{Ra}$ **[19]**  $\boldsymbol{CEBPAi=cPROT\cdot mCEBPA-CEBPAa}$ **[20]**  $\boldsymbol{NFKBi=cPROT\cdot mNFKB-NFKBa}$ **[21]**  $\boldsymbol{STAT}\boldsymbol{1i=cPROT\cdot mSTAT}\boldsymbol{1-STAT}\boldsymbol{1}\boldsymbol{a}$ **[22]**  $\boldsymbol{IRF}\boldsymbol{8=cPROT\cdot mIRF}\boldsymbol{8}$ **[23]**  $\boldsymbol{PU}\boldsymbol{1=cPROT\cdot mPU}\boldsymbol{1}$ **[24]**  $\boldsymbol{CSF}\boldsymbol{3}\boldsymbol{R=cPROT\cdot mCSF}\boldsymbol{3}\boldsymbol{R}$ **[25]**  $\boldsymbol{IL}\boldsymbol{6}\boldsymbol{R=cPROT\cdot mIL}\boldsymbol{6}\boldsymbol{R}$ **[26]**  $\boldsymbol{TNFR}\boldsymbol{1=cPROT\cdot mTNFR}\boldsymbol{1}$ **[27]**  $\boldsymbol{CSF}\boldsymbol{1=cPROT\cdot mCSF}\boldsymbol{1}$ **[28]** |
| --- |

**S1 Table C. Parameters of the mathematical model.**

| **Symbol of the parameter** | **Value of the parameter *** | **Definition of the parameter** |
| --- | --- | --- |
| *V*_M1IRF8_ | 0.0000014 | Basal transcription rate of IRF8 |
| *V*_M2IRF8_ | 0.00208 | Transcription rate constant of IRF8 regulated by PU1 |
| *V*_M3IRF8_ | 0.0104 | Transcription rate constant of IRF8 regulated by STAT1 |
| *K*_AS1PU1_ | 500 | Constant for activation of IRF8 synthesis by PU1 |
| *K*_STAT1A_ | 100 | Constant for activation of IRF8 synthesis by STAT1 |
| *LPS2* | 1 in the presence of LPS  0 in the absence of LPS | Induction of GRN components transcription by LPS |
| *LPSirf8* | 0.0005 | Induction of IRF8 transcription by LPS |
| *n* | 2 | Degree of cooperativity for the activation of transcription synthesis ** |
| *k*_DMIRF8_ | 0.1669 | Degradation rate constant of IRF8 mRNA |
| *V*_M1PU1_ | 0.000005 | Basal transcription rate of PU1 |
| *V*_M2PU1_ | 0.0005 | Transcription rate constant of PU1 regulated by PU1 |
| *V*_M3PU1_ | 0.002 | Transcription rate constant of PU1 regulated by CEBPA |
| *V*_M4PU1_ | 0.0005 | Transcription rate constant of PU1 regulated by NFKB |
| *K*_AS2PU1_ | 5000 | Constant for activation of PU1 synthesis by PU1 |
| *K*_AS1CEBPA_ | 10 | Constant for activation of PU1 synthesis by CEBPA |
| *K*_S5ANFKB_ | 6.67 | Constant for activation of PU1 synthesis by NFKB |
| *LPSpu1* | 0.002 | Induction of PU1 transcription by LPS |
| *k*_DMPU1_ | 0.095 | Degradation rate constant of PU1 mRNA |
| *V*_M1CSF3R_ | 0.000000067 | Basal transcription rate of CSF3R |
| *V*_M2CSF3R_ | 0.0000133 | Transcription rate constant of CSF3R regulated by PU1 |
| *V*_M3CSF3R_ | 0.00093 | Transcription rate constant of CSF3R regulated by CEBPA |
| *V*_M4CSF3R_ | 0.0001 | Transcription rate constant of CSF3R regulated by STAT3 |
| *K*_AS3PU1_ | 1000 | Constant for activation of CSF3R synthesis by PU1 |
| *K*_AS2CEBPA_ | 20 | Constant for activation of CSF3R synthesis by CEBPA |
| *K*_AS4STAT3A_ | 25 | Constant for activation of CSF3R synthesis by STAT3 |
| *LPScsf3r* | 0.00035 | Induction of CSF3R transcription by LPS |
| *k*_DMCSF3R_ | 0.136 | Degradation rate constant of CSF3R mRNA |
| *V*_M1IL6R_ | 0.00005 | Basal transcription rate of IL6R |
| *V*_M2IL6R_ | 0.005 | Transcription rate constant of IL6R regulated by CEBPA |
| *V*_M3IL6R_ | 0.00325 | Transcription rate constant of IL6R regulated by STAT3 |
| *V*_M4IL6R_ | 0.00025 | Transcription rate constant of IL6R regulated by STAT1 |
| *K*_AS3CEBPA_ | 10 | Constant for activation of IL6R synthesis by CEBPA |
| *K*_AS5CEBPA_ | 50 | Constant for activation of IL6R synthesis by STAT3 |
| *K*_AS6CEBPA_ | 62.5 | Constant for activation of IL6R synthesis by STAT1 |
| *LPSil6r* | 0 | Induction of IL6R transcription by LPS |
| *k*_DMIL6R_ | 0.15 | Degradation rate constant of IL6R mRNA |
| *V*_M1TNFR1_ | 0.00000125 | Basal transcription rate of TNFR1 |
| *V*_M2TNFR1_ | 0.0000375 | Transcription rate constant of TNFR1 regulated by STAT3 |
| *V*_M3TNFR1_ | 0.0015 | Transcription rate constant of TNFR1 regulated by CEBPA |
| *K*_ASSTAT3A_ | 50 | Constant for activation of TNFR1 synthesis by STAT3 |
| *K*_AS7CEBPA_ | 7.5 | Constant for activation of TNFR1 synthesis by CEBPA |
| *LPStnfr1* | 0.0011 | Induction of TNFR1 transcription by LPS |
| *k*_DMTNFR1_ | 0.075 | Degradation rate constant of TNFR1 mRNA |
| *V*_M1CSF1_ | 0.0000055 | Basal transcription rate of CSF1 |
| *V*_M2CSF1_ | 0.0028 | Transcription rate constant of CSF1 regulated by NFKB |
| *V*_M3CSF1_ | 0.095 | Transcription rate constant of CSF1 regulated by STAT1 |
| *V*_M4CSF1_ | 0.00055 | Transcription rate constant of CSF1 regulated by STAT3 |
| *V*_M5CSF1_ | 0.022 | Transcription rate constant of CSF1 regulated by PU1 |
| *K*_S1ANFKB_ | 66.67 | Constant for activation of CSF1 synthesis by NFKB |
| *K*_STAT1A2_ | 162.5 | Constant for activation of CSF1 synthesis by STAT1 |
| *K*_AS2STAT3A_ | 25 | Constant for activation of CSF1 synthesis by STAT3 |
| *K*_AS5PU1_ | 500 | Constant for activation of CSF1 synthesis by PU1 |
| *LPScsf1* | 0.005 | Induction of CSF1 transcription by LPS |
| *k*_DMCSF1_ | 0.13 | Degradation rate constant of CSF1 mRNA |
| *V*_M1STAT3_ | 0.00005 | Basal transcription rate of STAT3 |
| *V*_M2STAT3_ | 0.00085 | Transcription rate constant of STAT3 regulated by STAT3 |
| *K*_STAT3A_ | 15 | Constant for activation of STAT3 synthesis by STAT3 |
| *LPSstat3* | 0.001 | Induction of STAT3 transcription by LPS |
| *k*_DMSTAT3_ | 0.097 | Degradation rate constant of STAT3 mRNA |
| *V*_M1CSF1R_ | 0.000286 | Basal transcription rate of CSF1R |
| *V*_M2CSF1R_ | 0.00143 | Transcription rate constant of CSF1R regulated by PU1 |
| *V*_M3CSF1R_ | 0.0143 | Transcription rate constant of CSF1R regulated by CEBPA |
| *V*_M4CSF1R_ | 0.000857 | Transcription rate constant of CSF1R regulated by STAT3 |
| *K*_AS4PU1_ | 3000 | Constant for activation of CSF1R synthesis by PU1 |
| *K*_AS4CEBPA_ | 20 | Constant for activation of CSF1R synthesis by CEBPA |
| *K*_AS5STAT3A_ | 25 | Constant for activation of CSF1R synthesis by STAT3 |
| *LPScsf1r* | 0.0017 | Induction of CSF1R transcription by LPS |
| *k*_DMCSF1R_ | 0.03 | Degradation rate constant of CSF1R mRNA |
| *V*_M1CEBPA_ | 0.00015 | Basal transcription rate of CEBPA |
| *V*_M2CEBPA_ | 0.00075 | Transcription rate constant of CEBPA regulated by PU1 |
| *V*_M3CEBPA_ | 0.0015 | Transcription rate constant of CEBPA regulated by STAT3 |
| *K*_AS10PU1_ | 500 | Constant for activation of CEBPA synthesis by PU1 |
| *K*_AS3STAT3A_ | 25 | Constant for activation of CEBPA synthesis by STAT3 |
| *K*_2LPS_ | 0.000000000001 | Constant for inhibition of CEBPA synthesis by LPS |
| *k*_DMCEBPA_ | 0.175 | Degradation rate constant of CEBPA mRNA |
| *V*_M1NFKB_ | 0.00000067 | Basal transcription rate of NFKB |
| *V*_M2NFKB_ | 0.0027 | Transcription rate constant of NFKB regulated by NFKB |
| *V*_M3NFKB_ | 0.02 | Transcription rate constant of NFKB regulated by LPS |
| *V*_M4NFKB_ | 0.00067 | Transcription rate constant of NFKB regulated by PU1 |
| *V*_M5NFKB_ | 0.0027 | Transcription rate constant of NFKB regulated by CEBPA |
| *K*_S3ANFKB_ | 6.67 | Constant for activation of NFKB synthesis by NFKB |
| *K*_LPS_ | 0.1 | Constant for activation of NFKB synthesis by LPS |
| *K*_AS6PU1_ | 5000 | Constant for activation of NFKB synthesis by PU1 |
| *K*_AS8CEBPA_ | 25 | Constant for activation of NFKB synthesis by CEBPA |
| *k*_DMNFKB_ | 0.137 | Degradation rate constant of NFKB mRNA |
| *V*_M1STAT1_ | 0.00000125 | Basal transcription rate of STAT1 |
| *V*_M2STAT1_ | 0.000625 | Transcription rate constant of STAT1 regulated by STAT1 |
| *V*_M3STAT1_ | 0.0025 | Transcription rate constant of STAT1 regulated by NFKB |
| *K*_STAT1A3_ | 6.25 | Constant for activation of STAT1 synthesis by STAT1 |
| *K*_S4ANFKB_ | 0.67 | Constant for activation of STAT1 synthesis by NFKB |
| *LPSstat1* | 0.015 | Induction of STAT1 transcription by LPS |
| *K_IRF8_* | 20.83 | Constant for inhibition of STAT1 synthesis by IRF8 |
| *k*_DMSTAT1_ | 0.1 | Degradation rate constant of STAT1 mRNA |
| *V*_A1STAT3_ | 1.6 | Rate constant for activation of STAT3 by TNFR1 |
| *V*_A2STAT3_ | 1.6 | Rate constant for activation of STAT3 by STAT1 |
| *V*_A3STAT3_ | 30 | Rate constant for activation of STAT3 by GCSFR |
| *V*_A4STAT3_ | 0.7 | Rate constant for activation of STAT3 by CSF1R |
| *K*_ASTAT3_ | 100 | Constant for activation of STAT3 protein |
| *K*_ISTAT3_ | 100 | Constant for inactivation of STAT3 protein |
| *V*_ISTAT3_ | 0.5 | Rate constant for inactivation of STAT3 protein |
| *V*_ACSF1R_ | 288 | Rate constant for activation of CSF1R by CSF1 |
| *K*_ACSF1R_ | 100 | Constant for activation of CSF1R protein |
| *V*_ICSF1R_ | 0.5 | Rate constant for inactivation of CSF1R protein |
| *K*_ICSF1R_ | 100 | Constant for inactivation of CSF1R protein |
| *V*_ACEBPA_ | 1050 | Rate constant for activation of CEBPA by CSF3R |
| *K*_ACEBPA_ | 1000 | Constant for activation of CEBPA protein |
| *V*_ICEBPA_ | 0.7 | Rate constant for inactivation of CEBPA protein |
| *K*_ICEBPA_ | 1000 | Constant for inactivation of CEBPA protein |
| *V*_ANFKB_ | 160 | Rate constant for activation of NFKB by TNFR1 |
| *K*_ANFKB_ | 500 | Constant for activation of NFKB protein |
| *V*_INFKB_ | 1 | Rate constant for inactivation of NFKB protein |
| *K*_INFKB_ | 1000 | Constant for inactivation of NFKB protein |
| *V*_A1STAT1_ | 2 | Rate constant for activation of STAT1 by IL6R |
| *V*_A2STAT1_ | 0.35 | Rate constant for activation of STAT1 by MCSFR |
| *V*_A3STAT1_ | 2 | Rate constant for activation of STAT1 by STAT3 |
| *V*_A4STAT1_ | 0.01 | Rate constant for activation of STAT1 by LPS |
| *K*_ASTAT1_ | 3000 | Constant for activation of STAT1 protein |
| *V*_ISTAT1_ | 0.06 | Rate constant for inactivation of STAT1 protein |
| *K*_ISTAT1_ | 3000 | Constant for inactivation of STAT1 protein |
| *cPROT* | 3000 | Ratio factor between mRNA and protein expression |
| *V*_SLPS_ | Square wave (20 or 0) | Accumulation rate of LPS |
| *k*_DLPS_ | 2 | Degradation rate constant of LPS |

^*^ The dynamic variables of the mathematical model are expressed relative to the housekeeping gene *ACTIN* (*ACTB*) and are thus dimensionless. The time unit of the rate constants is inverse hour (h^-1^).

** We consider n = 2 for components that can form dimers. Considering a degree of cooperativity with n = 4 or n = 8 would artificially increase the nonlinearity of the model.

**S1 Table D. Initial conditions of the model.**

| **Variable** | **Equation** | **Value of the initial condition** |
| --- | --- | --- |
| *mIRF8* | 1 | 0.01 |
| *mPU1* | 2 | 0.04 |
| *mCSF3R* | 3 | 0.0005 |
| *mIL6R* | 4 | 0.004 |
| *mTNFR1* | 5 | 0.01 |
| *mCSF1* | 6 | 0.1 |
| *mSTAT3* | 7 | 0.004 |
| *mCSF1R* | 8 | 0.025 |
| *mCEBPA* | 9 | 0.003 |
| *mNFKB* | 10 | 0.005 |
| *mSTAT1* | 11 | 0.007 |
| *STAT3a* | 12 | 15 |
| *CSF1Ra* | 13 | 15 |
| *CEBPAa* | 14 | 15 |
| *NFKBa* | 15 | 15 |
| *STAT1a* | 16 | 15 |
| *LPS* | 17 | 20 |

**S1 Table E. Half-life durations of the network components.**

| **Component** | **Experimental**  **mRNA half-life (h)** | ***In silico***  **mRNA half-life (h)** | **Reference** |
| --- | --- | --- | --- |
| IRF8 | 4.1 (0 – 6.7) | 4.1 | [1,2] |
| PU1 | 7.3 (0 – 24) | 7.3 | [1,2] |
| CSF3R | 5.1 (3.4 – 6.2) | 5.1 | [1,2] |
| IL6R | 12.1 (0 – 24) | 4.6 | [1,2] |
| TNFR1 | 9.2 (6.1 – 24) | 9.2 | [1,2]] |
| CSF1 | 5.2 (0 – 6.5) | 5.3 | [1,2] |
| STAT3 | 7.1 (4 – 9.5) | 6.9 | [1,2] |
| CSF1R | 24 | 23.1 | [1,2] |
| CEBPA | 3.9 (3.4 – 8.6) | 4.0 | [1,2] |
| NFKB | 5.1 (3.6 – 6.9) | 5.1 | [1,2] |
| STAT1 | 7.0 (5.5 – 11) | 6.9 | [1,2] |

References

1. Schwanhausser B, Busse D, Li N, Dittmar G, Schuchhardt J, Wolf J, et al. Global quantification of mammalian gene expression control. Nature. 2011;473(7347):337-42. doi: 10.1038/nature10098. PubMed PMID: 21593866.

2. Sharova LV, Alexei A, Sharov AA, Nedorezov T, Piao Y, Shaik N, Ko MSH. Database for mRNA Half-Life of 19 977 Genes Obtained by DNA Microarray Analysis of Pluripotent and Differentiating Mouse Embryonic Stem Cells. DNA Research. 2009; 16, (1):45–58. doi:10.1093/dnares/dsn030. PMID: 19001483
