# Supplementary figures and images for: Temporal dynamics of a CSF1R signaling gene regulatory network involved in epilepsy
Source: PLoS Comput Biol. 2021 Apr 5;17(4):e1008854. doi: 10.1371/journal.pcbi.1008854 (PMC8057615; doi:10.1371/journal.pcbi.1008854)

**A**

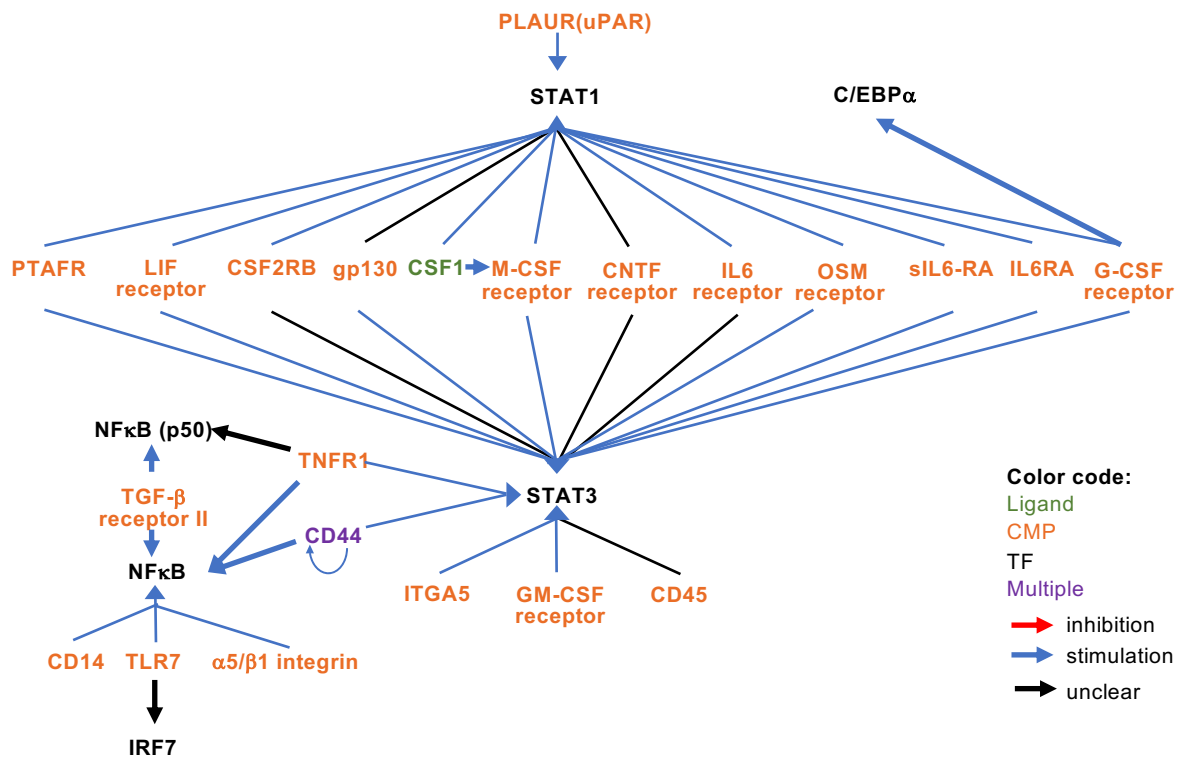

**B**

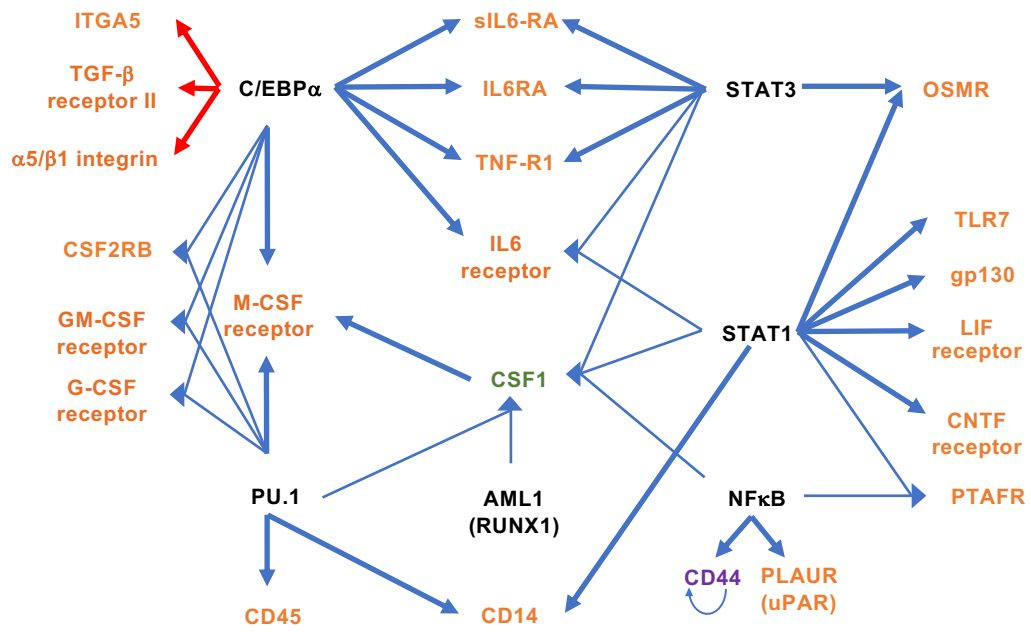

**C**

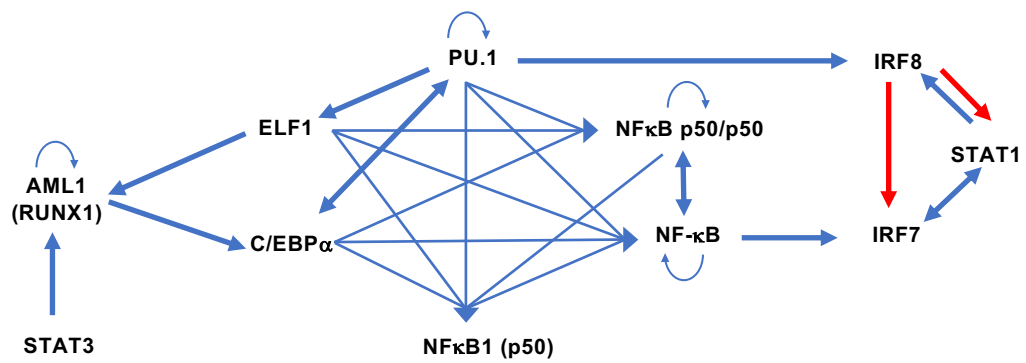

Supplement: S1 Fig — Each arrow represents an interaction. The network has been divided in 3 sub-plots for clarity: (A) CMP→TF and ligand→CMP interactions; (B) TF→ligand and TF→CMP interactions; (C) TF→TF interactions. Interactions between CMP and TF occur via a signaling pathway. Interactions from a TF correspond to a transcriptional control. Interaction from ligand to CMP correspond to the activation or inhibition of the CMP by its ligand. Color code is indicated. (PDF) [file pcbi.1008854.s003.pdf]

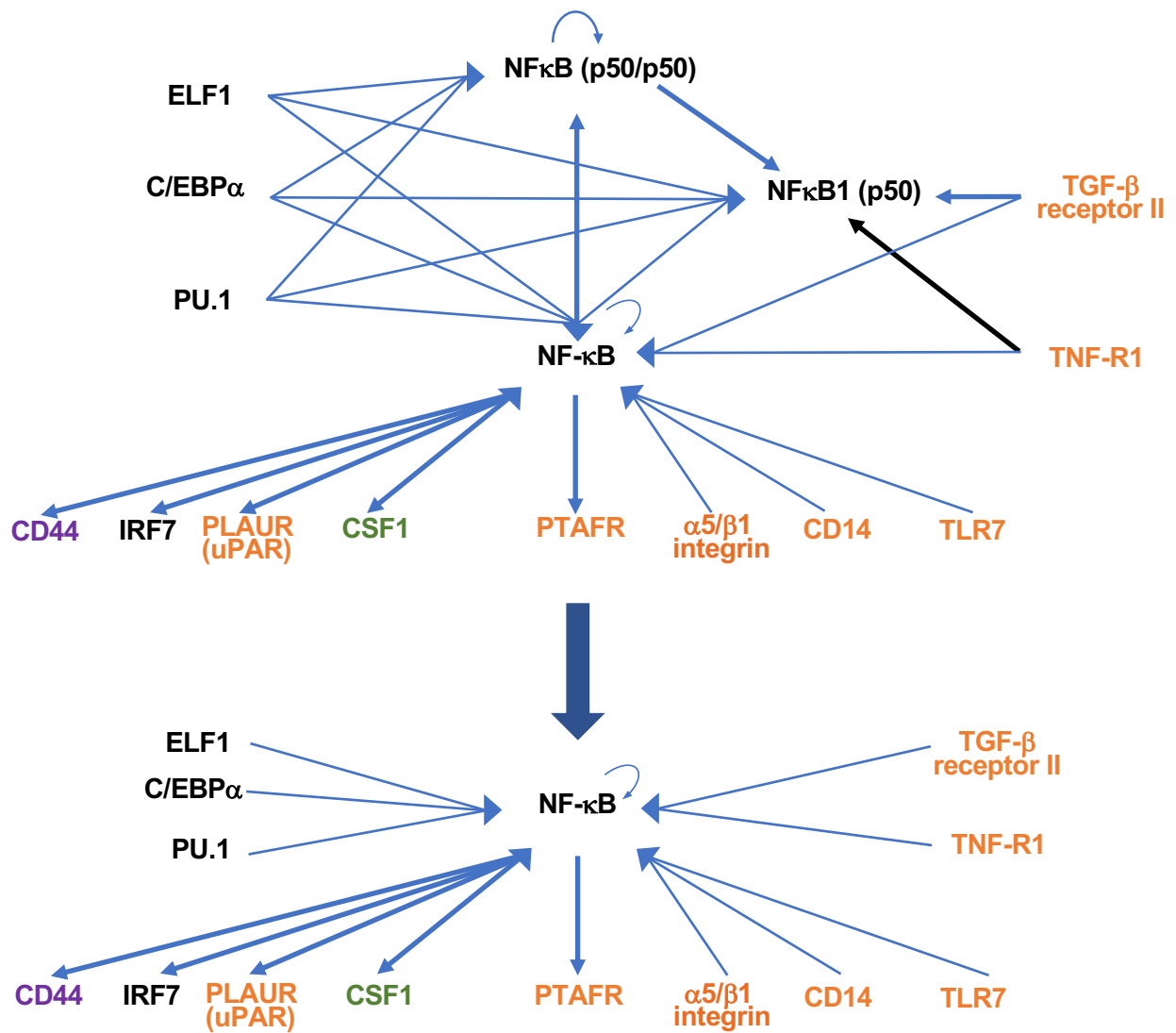

Supplement: S2 Fig — NF-κB1 (p50) and NF-κB p50/p50 are precursors of NF-κB, and all upstream regulators of these precursors also regulate NF-κB. Therefore, the 2 precursors were removed. TNF-R1 activates NF-κB and has a conflicting effect on precursor NF-κB1 (p50): the well documented direct effect of NF-κB activation by TNF-R1 was kept. (PDF) [file pcbi.1008854.s004.pdf]

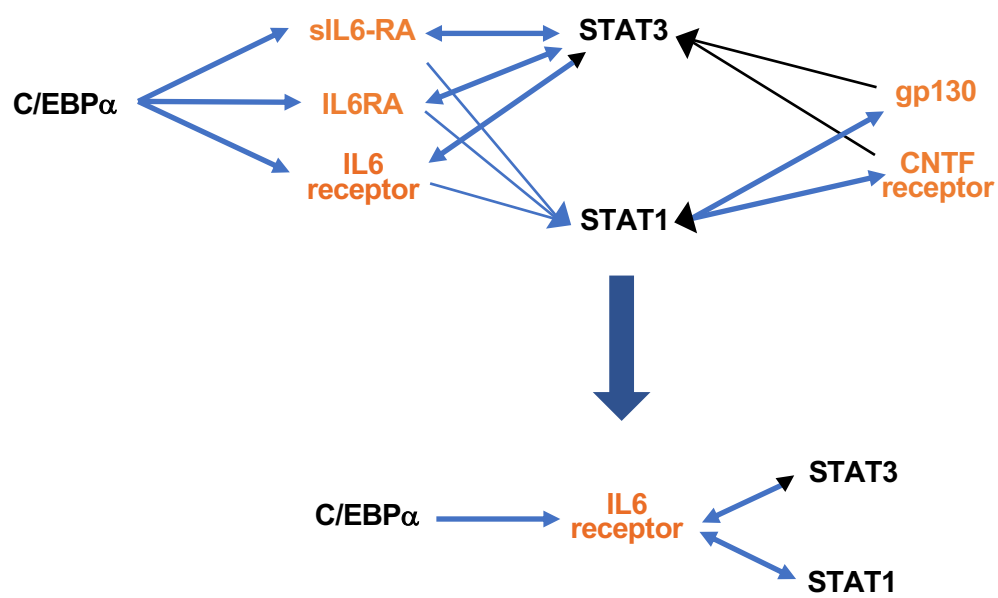

Supplement: S3 Fig — sIL6RA could be removed since sIL6RA is the soluble form of IL6RA, and both share the same interactions with the rest of the network. CNTF receptor is a component of gp130 and they both share the same interactions with the rest of the network; therefore CNTF receptor was removed. In addition, gp130 and IL6RA/sIL6RA are components of IL-6 receptor. All regulators of gp130 or IL6RA also regulate the IL-6 receptor. The network was thus simplified by removing IL6RA, sIL6RA and gp130. IL-6 receptor activates STAT1, while its components gp130/CNTF receptors have an unclear effect on the same TF. Again, the most direct regulation was kept, namely STAT1 activation by IL-6 receptor. (PDF) [file pcbi.1008854.s005.pdf]

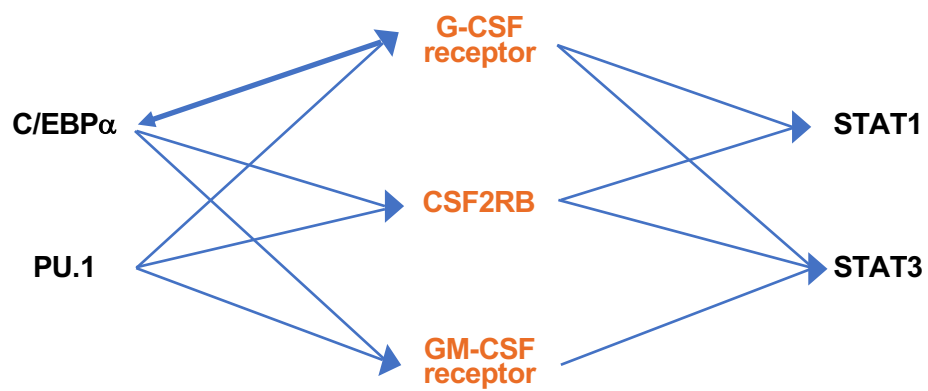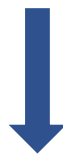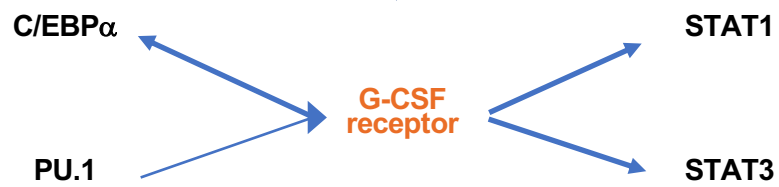

Supplement: S4 Fig — CSF2RB is a subunit of GM-CSF receptor, and its interactions are covered by the latter. Therefore CSF2RB was filtered out. In addition, G-CSF and GM-CSF receptors are similar and share the same interactions within the network. The only exception is the additional activation of C/EBPα by G-CSF receptor (and not by GM-CSF receptor). Therefore, only G-CSF (CSF3R) receptor was maintained in the network. (PDF) [file pcbi.1008854.s006.pdf]

A

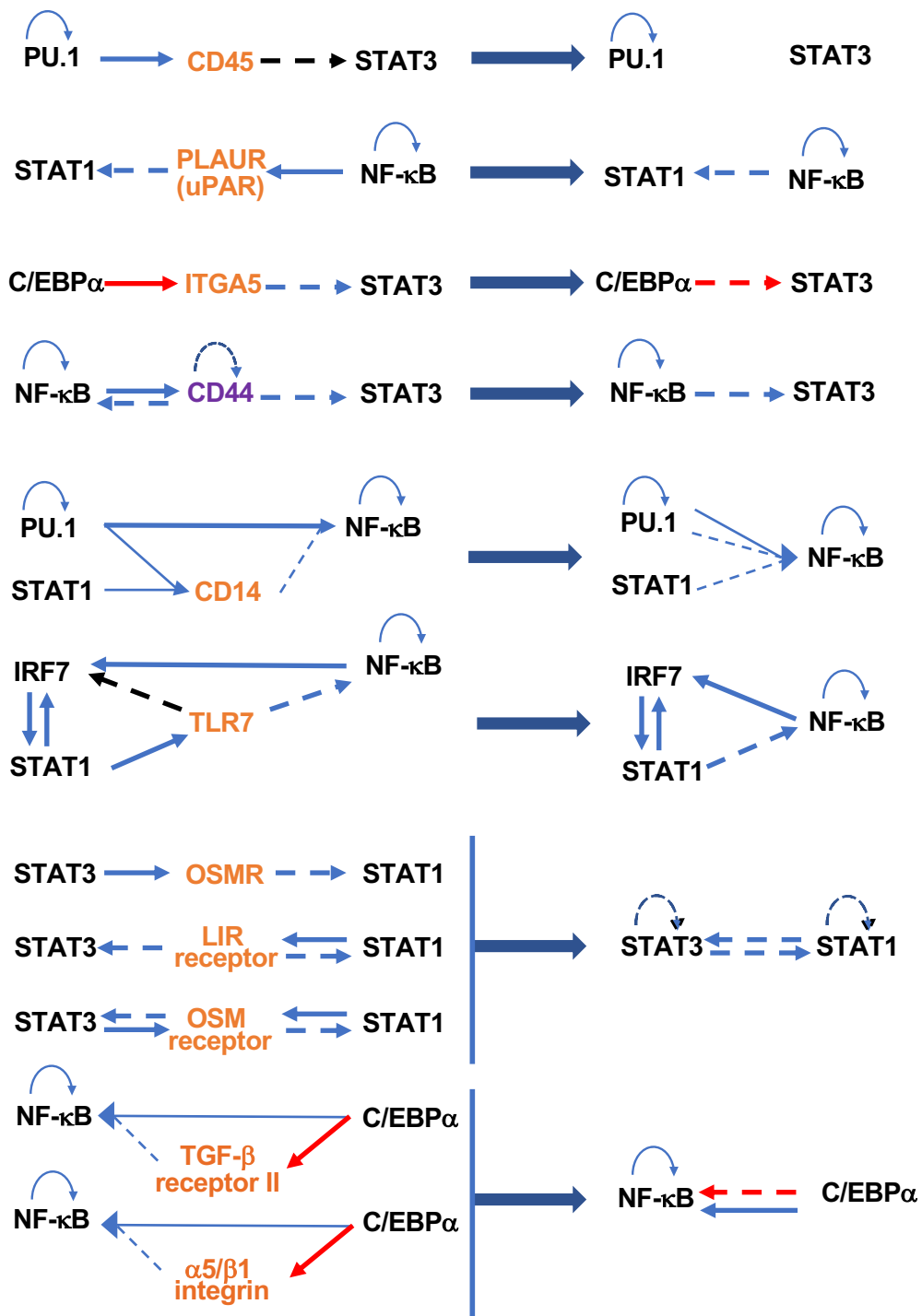

B

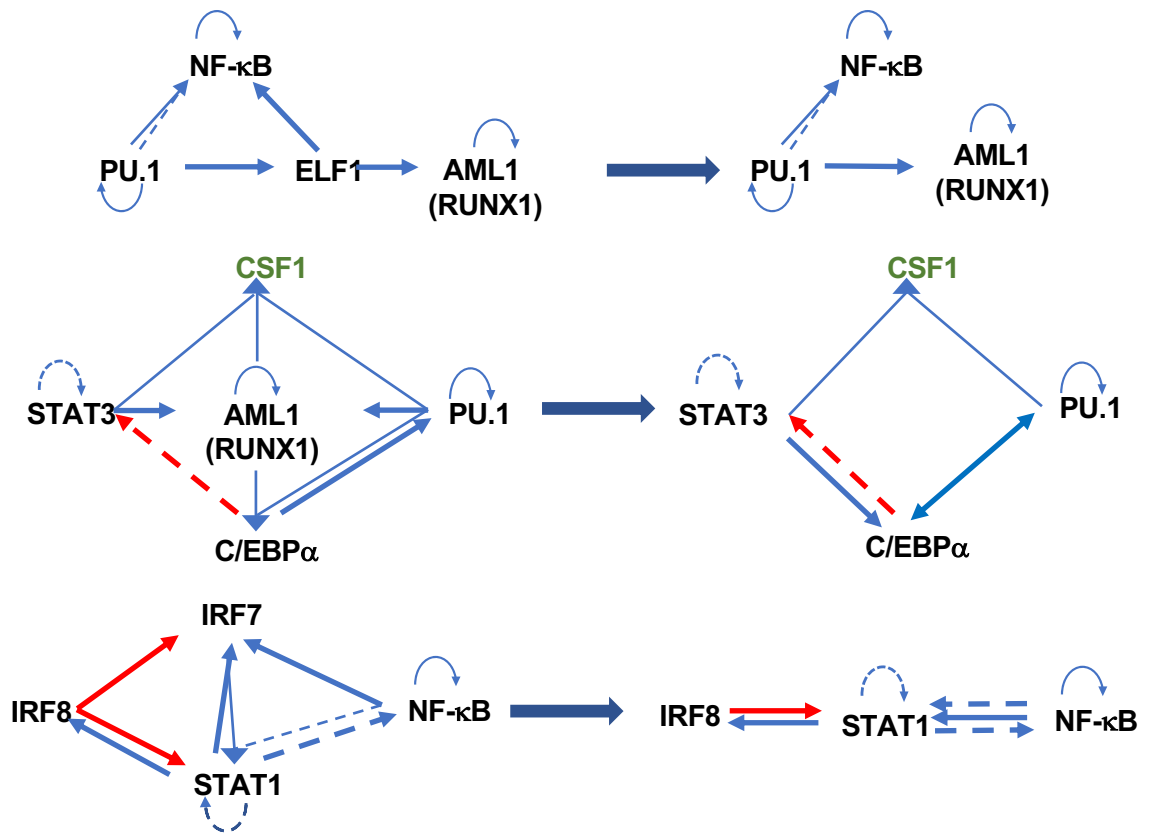

C

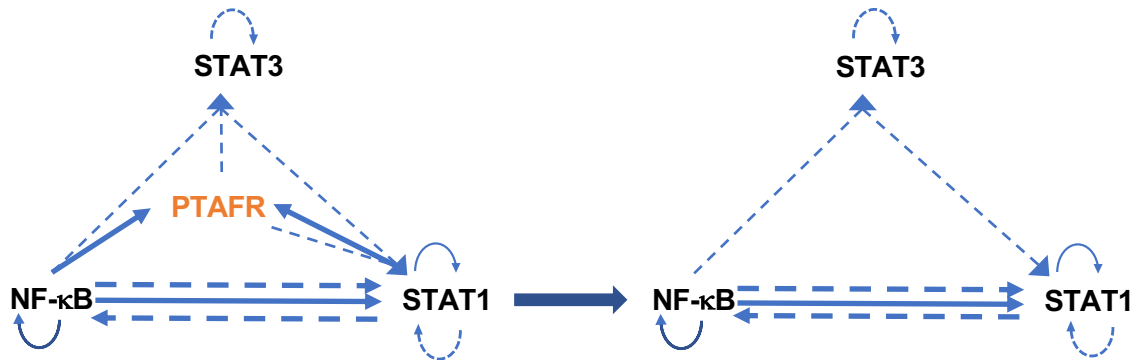

Supplement: S5 Fig — (A) In each case presented in this figure, a CMP could be removed, while considering the sum of interactions between the remaining nodes. In this process, new unclear interactions are not kept in the network. Dashed lines, regulation of protein function; plain lines, transcriptional regulation. (B) Three sequential steps were followed after removal of CMPs. At each step, the TF could be removed, while considering the sum of interactions between the remaining nodes. (C) PTAFR can be removed without adding any interaction, as all the interactions it mediates are already present. (PDF) [file pcbi.1008854.s007.pdf]

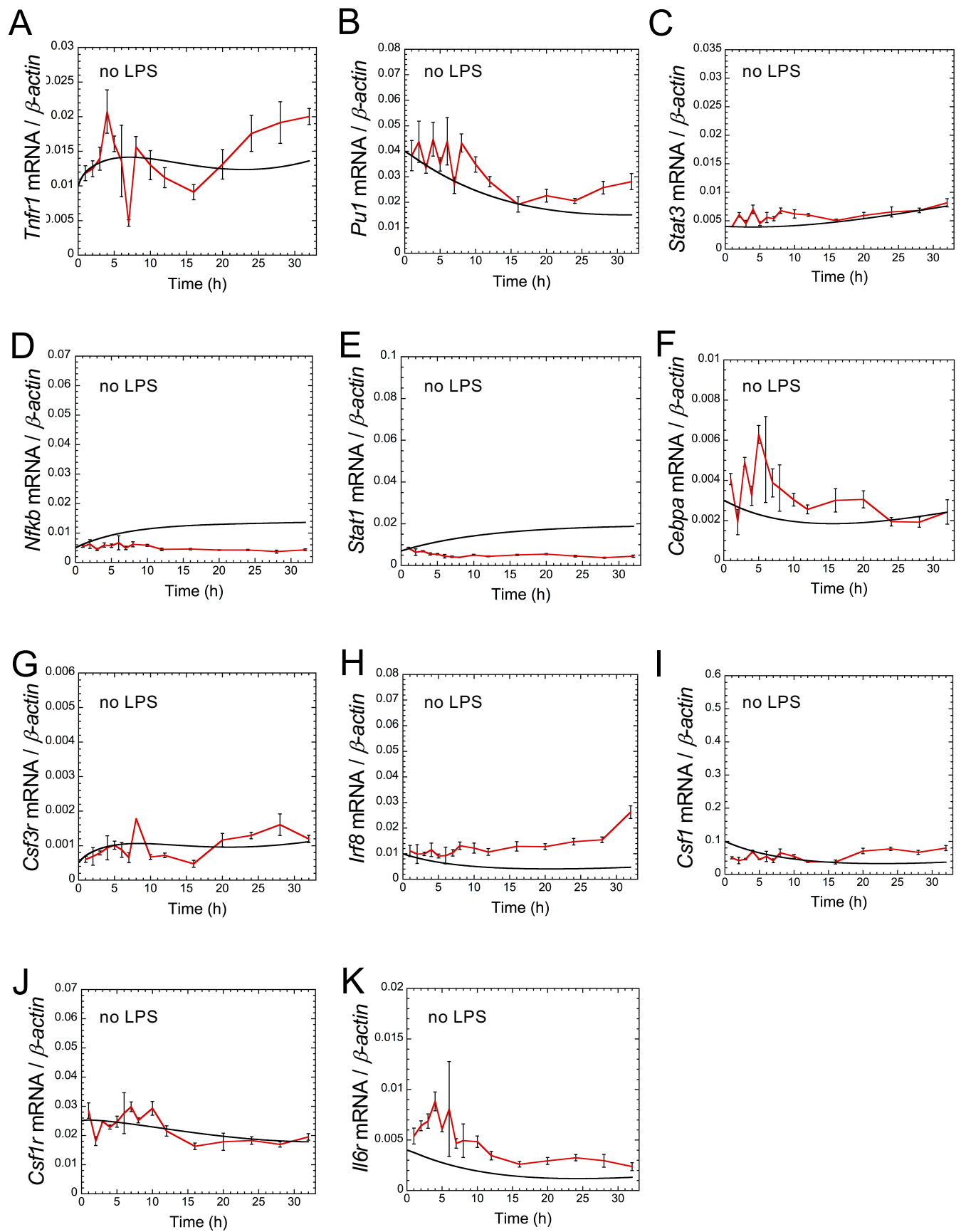

Supplement: S6 Fig — Temporal evolution of the mRNA expression level of (A) Tnfr1, (B) Pu1, (C) Stat3, (D) Nfkb, (E) Stat1, (F) Cebpa, (G) Csf3r, (H) Irf8, (I) Csf1, (J) Csf1r and (K) Il6r. Red curves: experimental data, black curves: mathematical model. Experimental data are the means (relative to β-actin) +/- SD; n = 4 and are collected at the indicated time points in BV-2 cells (S5–S6 Data). Conditions of the numerical simulations are outlined in S1 Text and Tables A-E in S1 Tables. (PDF) [file pcbi.1008854.s008.pdf]

**A**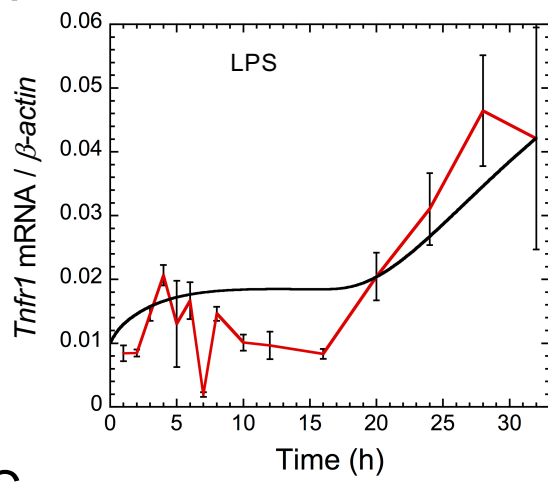**B**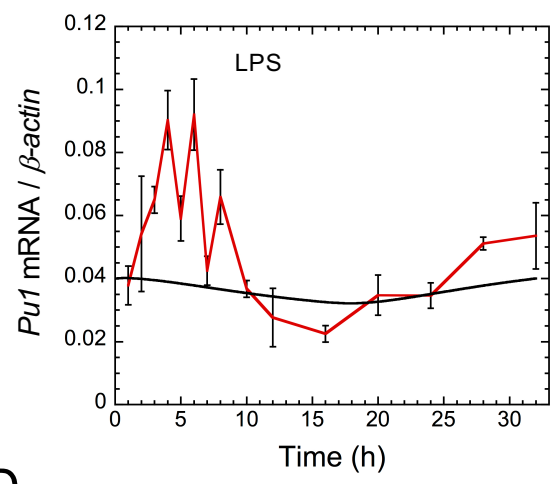**C**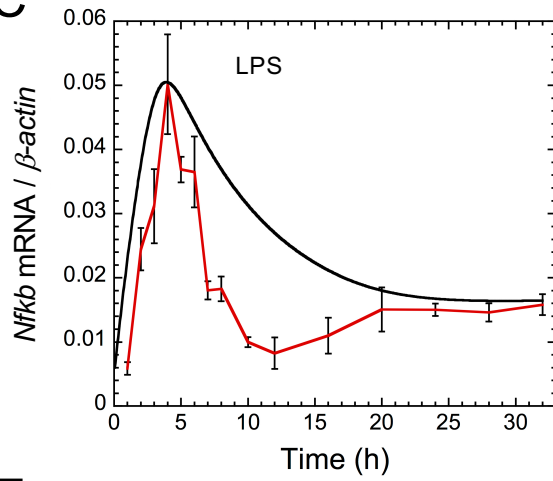**D**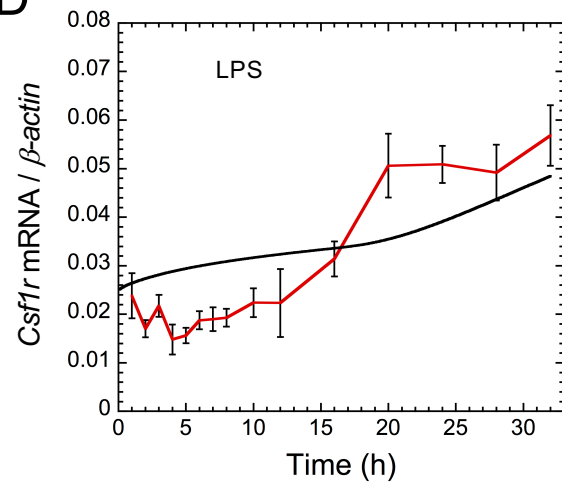**E**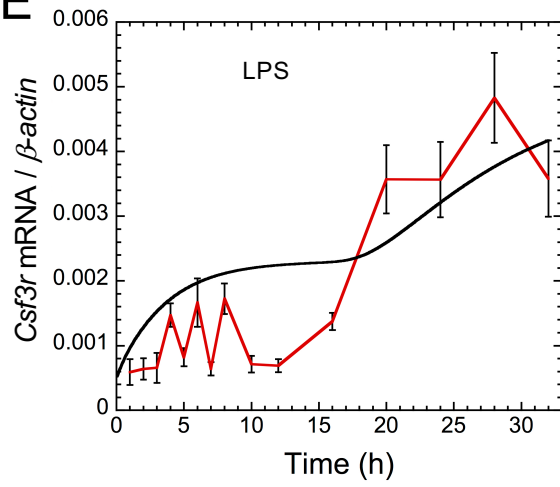

Supplement: S7 Fig — Temporal evolution of the mRNA expression levels of (A) Tnfr1, (B) Pu1, (C) Nfkb, (D) Csf1r and (E) Csf3r. LPS = 1 μM added at t = 0h. Red curves: experimental data, black curves: mathematical model. Experimental data are the means (relative to β-actin) +/- SD; n = 4 and are collected at the indicated time points after LPS administration in BV-2 cells (S7–S8 Data). Conditions of the numerical simulations are outlined in S1 Text and Tables A-E in S1 Tables. (PDF) [file pcbi.1008854.s009.pdf]

A

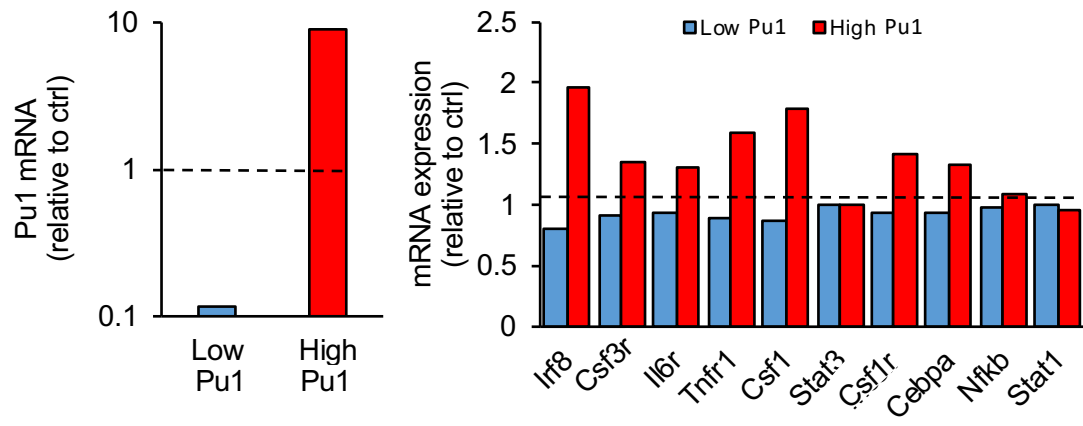

B

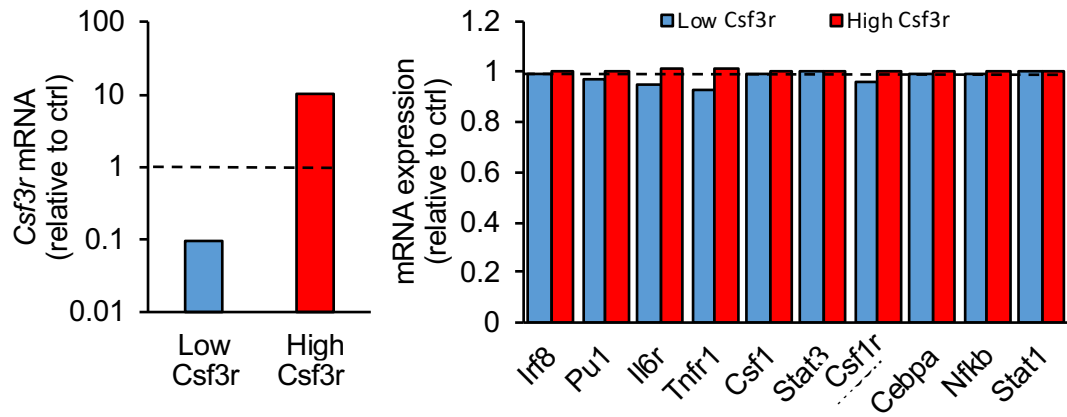

C

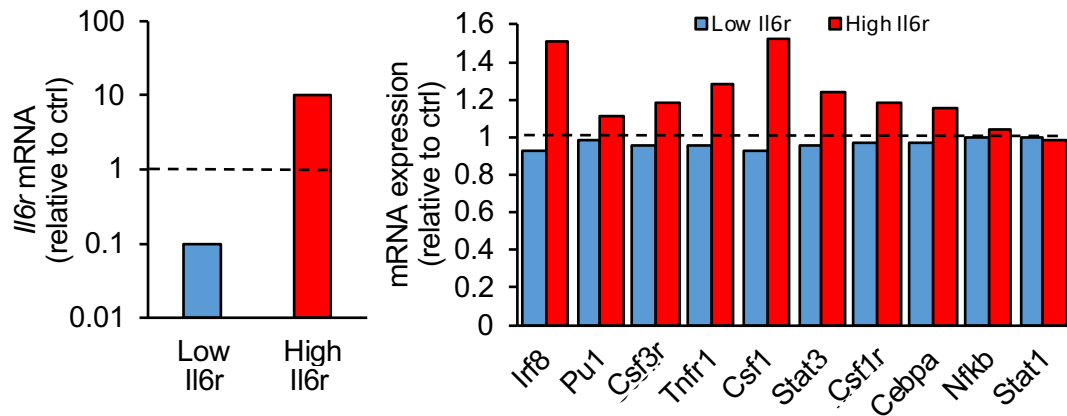

D

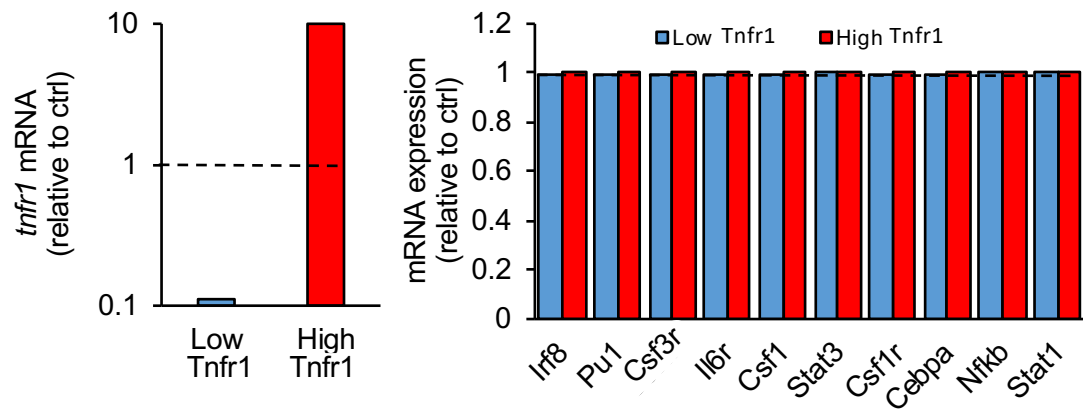

Supplement: S8 Fig — Impact of low (blue bars) or high expression levels (red bars) of (A) Pu1, (B) Csf3r, (C) Il6r and (D) Tnfr1 on the mRNA expression levels of the other GRN components. The control conditions in the simulations (horizontal dashed lines) are the expression levels of the GRN components in the absence of LPS after 48h. Numerical simulations for the various conditions are described in S1 Text and Tables A-E in S1 Tables. (PDF) [file pcbi.1008854.s010.pdf]

**A**

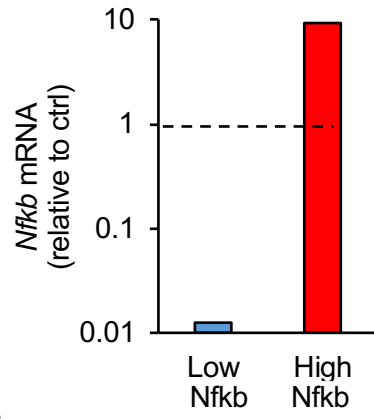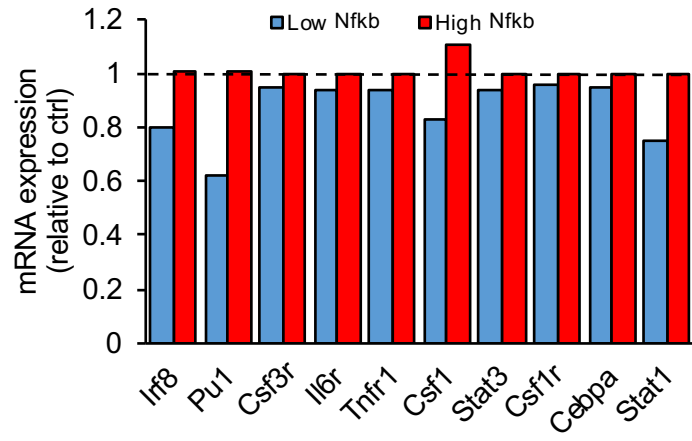

**B**

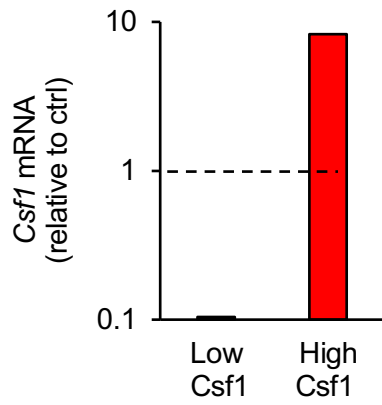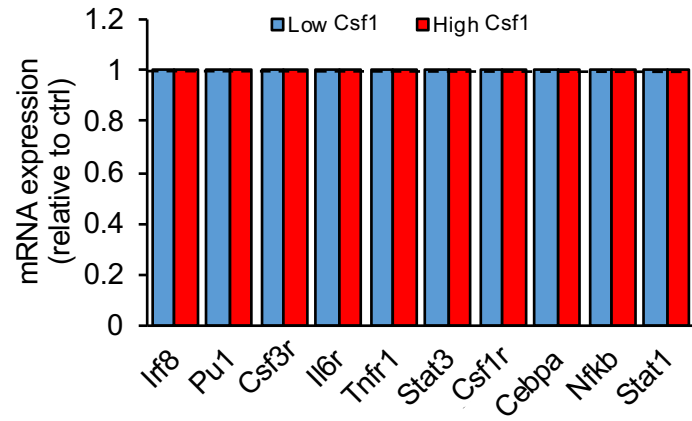

**C**

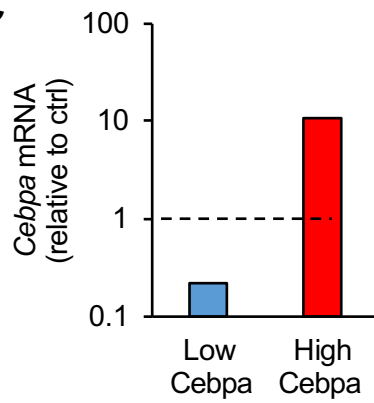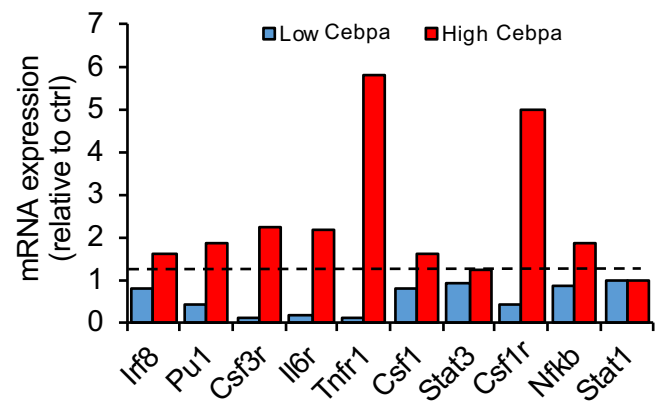

Supplement: S9 Fig — Impact of low (blue bars) or high expression levels (red bars) of (A) NfkB, (B) Csf1 and (C) Cebpa on the mRNA expression levels of the other GRN components. The control conditions in the simulations (horizontal dashed lines) are the expression levels of the GRN components in the absence of LPS after 48h. Numerical simulations for the various conditions are found in S1 Text and Tables A-E in S1 Tables. (PDF) [file pcbi.1008854.s011.pdf]

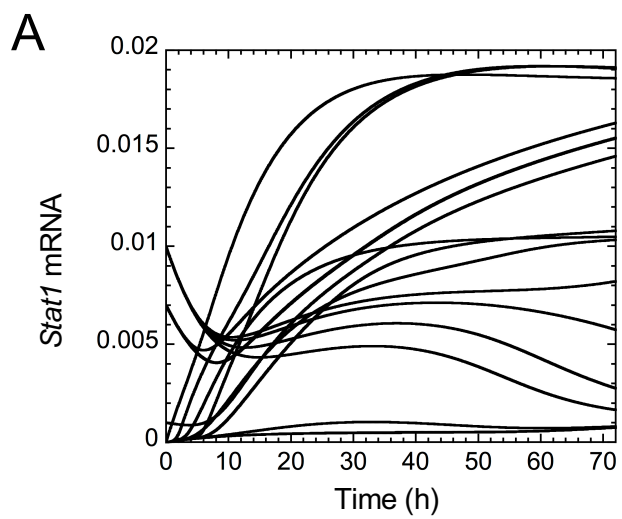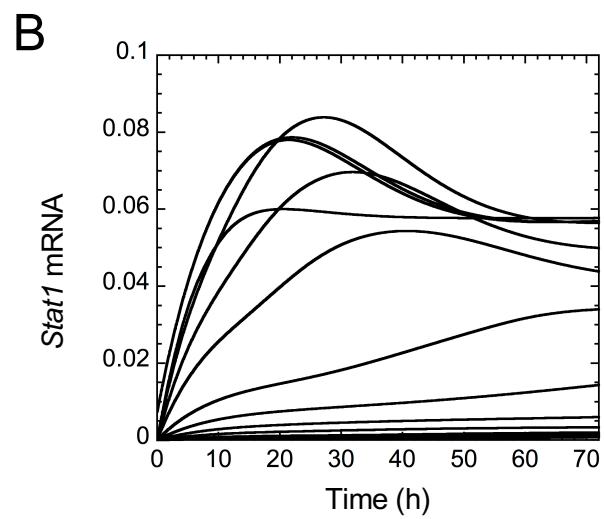

Supplement: S10 Fig — Temporal evolution of Stat1 mRNA in the absence (A) or presence (B) of LPS, using different initial conditions randomly chosen between the values of 0 and 20. Simulations were performed with XPPAUT. Parameter values are as in Table C in S1 Tables. (PDF) [file pcbi.1008854.s012.pdf]
